# A first phylogenetic study on stoloniferous octocorals off the coast of Kota Kinabalu, Sabah, Malaysia, with the description of two new genera and five new species

**DOI:** 10.3897/zookeys.872.36288

**Published:** 2019-08-26

**Authors:** Yee Wah Lau, James D. Reimer

**Affiliations:** 1 Molecular Invertebrate Systematics and Ecology Laboratory, Graduate School of Engineering and Science, University of the Ryukyus, 1 Senbaru, Nishihara, Okinawa 903-0213, Japan University of the Ryukyus Nishihara Japan; 2 Tropical Biosphere Research Center, University of the Ryukyus, 1 Senbaru, Nishihara, Okinawa 903-0213, Japan University of the Ryukyus Nishihara Japan

**Keywords:** 28S rDNA, Arulidae, biodiversity, Clavulariidae, COI, Coral Triangle, mtMutS, ND6, Stolonifera, systematics, TARP, taxonomy

## Abstract

Sabah, Malaysia, is well known for its extensive and diverse coral reefs. It is located on the northwestern edge of the Coral Triangle, the region with the highest marine biodiversity. Much of the marine fauna here is still unknown, especially inconspicuous animals, such as small stoloniferous octocorals, which are common on coral reefs. Here, we describe two new monospecific genera of the family Arulidae found off the coast of Kota Kinabalu, Sabah, East Malaysia; *Bunga payung***gen. nov. et sp. nov.** and *Laeta waheedae***gen. nov. et sp. nov.** As well, the stoloniferan genus *Phenganax* Alderslade & McFadden, 2011 belonging to the family Clavulariidae is expanded with three new species, *P. marumi***sp. nov.**, *P. subtilis***sp. nov.**, and *P. stokvisi***sp. nov.**, which are all sclerite-free. Additionally, we report a possibly undescribed species, closely related to the clavulariid genera *Azoriella* Lopez-Gonzalez & Gili, 2001 and *Cervera* Lopez-Gonzalez et al., 1995. As this and other recent studies have shown, discoveries of small stoloniferous octocorals are helping to fill gaps in our knowledge of the overall systematics of Octocorallia.

## Introduction

Coral reefs fringe one-sixth of the world’s coastlines and constitute the most biologically diverse shallow-water marine ecosystems, supporting thousands of species (Birkeland 1997; Reaka-Kudla 1997; Roberts et al. 2002). The estimated global biodiversity of coral reefs is approximately 950,000 (±40%) species, of which 90% are not yet described (Fisher et al. 2015). One reason marine biodiversity is globally underestimated is that there are many small and cryptic species that are usually overlooked in diversity assessments (Wolf et al. 1983; Hoeksema 2017; Lau et al. 2018, 2019; Sabroux et al. 2019). An estimate from 2002 indicated that 25% of the world’s coral reefs have already been severely damaged due to global warming (Goreau et al. 2000; Roberts et al. 2002), pollution, and destructive fisheries methods (dynamite and poison) (Polunin and Roberts 1996; Roberts et al. 2002; Hughes et al. 2017; Heery et al. 2018). Damage continues to accumulate (Hughes et al. 2018), thus threatening coral reef biodiversity and possibly causing the extinction of organisms that have not been scientifically described yet (Carpenter et al. 2008; Hoeksema 2017).

The highest concentrations of coral reef species can be found in the Coral Triangle in the Central Indo-Pacific (Hoeksema 2007, 2017). Continuing analyses have resulted in the boundaries of the Coral Triangle biodiversity hotspot being refined over time (Hoeksema 2007; Huang et al. 2015; Veron et al. 2015; Lane and Hoeksema 2016). The coastline of Sabah, on the northern tip of East Malaysia, is located within the area that makes up the outer western edge of the Coral Triangle (Hoeksema 2007; Waheed and Hoeksema 2013, 2014; Waheed et al. 2015a). Malaysia is known for its extensive coral reefs that cover an area of approximately 4000 km^2^ with the majority (>75%) situated in Sabah (Burke et al. 2002; Waheed et al. 2007). Sabah (with its offshore islands) has an extensive history of marine research, focusing mainly on scleractinian diversity and coral cover (Lulofs 1973; Lulofs et al. 1974; Wood 1977, 1979; Mathias and Langham 1978; Nyanti and Johnston 1992; Pilcher and Cabanban 2000; Waheed and Hoeksema 2013, 2014; Reef Check Malaysia 2014, Waheed et al. 2015a, 2015b), and reef fish (Kassem et al. 2012; Reef Check Malaysia 2014; Townsend 2015).

Just off the coast of Kota Kinabalu, the capital of Sabah, there is an assemblage of five islands that make up Tunku Abdul Rahman Park (TARP): Gaya, Manukan, Sapi, Sulug, and Mamutik islands cover an area of approximately 50 km^2^, including their surrounding reefs and sea (Spait 2001; Reef Check Malaysia 2014; Waheed and Hoeksema 2014). TARP has been protected since 1977 and is reserved and managed by Sabah Parks. Despite the protection of the coral reefs in the park against destructive fishing and land-based developments (Spait 2001; Waheed et al. 2007), the coral cover in the park has seen a severe decline since 1994 owing to continued anthropogenic influences and a major crown-of-thorns starfish outbreak (Praveena et al. 2012; Waheed and Hoeksema 2014; Heery et al. 2018), emphasizing the urgent need for diversity research and taxonomic documentation.

For the shallow waters of the Indo-Pacific, over 100 genera in 23 families of Alcyonacea (e.g., Alderslade 2001, 2002; Fabricius and Alderslade 2001; Benayahu et al. 2004; van Ofwegen 2005), nine genera in five families of Pennatulacea (Williams 1996), and two genera in two families of Helioporacea have been described (Fabricius et al. 2007; Miyazaki and Reimer 2015). However, compared to scleractinian corals, octocorals have received little research attention and are less well documented (Fabricius and Alderslade 2001; Breedy and Cortés 2008; McFadden et al. 2010; Samimi-Namin and Van Ofwegen 2012). Despite Sabah’s location within the area that makes up the outer western edge of the Coral Triangle (Hoeksema 2007; Waheed and Hoeksema 2014; Waheed et al. 2015a), and the fact that octocorals are an abundant and species-rich group on Indo-Pacific coral reefs, relatively little research has been done on octocorals in Sabah (Kassem et al. 2012).

Zooxanthellate octocorals are, however, similarly affected by global climate change and other anthropogenic and natural threats (Prada et al. 2010; Dias et al. 2016; Van de Water et al. 2018). Additionally, octocorals are one of the most widely distributed and common benthic groups, occurring from shallow tropics to the Antarctic deep sea, and are important members of the benthic community, providing refuge and habitat for numerous organisms (Sánchez et al. 2003; Sánchez 2016). Due to their abundance, diverse structural complexity, and symbiotic relationships, octocorals play an important role in the energy transfer between plankton and other benthos (Van de Water et al. 2018) and should receive more research attention. However, research focused on octocorals is still uncommon, and the importance of their taxonomy is underestimated (McFadden and Ofwegen 2012; Benayahu et al. 2017; Conti-Jerpe and Freshwater 2017). This is particularly true for one group of octocorals, namely species of the subordinal group Stolonifera, many of which are inconspicuous, with small polyps (often ~2–3 mm in diameter) and colonies, and are hard to find, one reason for the scant amount of information available for this group (Wolf et al. 1983; McFadden and Ofwegen 2012). In the TARP area, next to sponges, algae, and hard corals, soft corals are the most dominant benthic fauna (Reef Check Malaysia 2014), however, research focusing on octocorals is completely lacking here. Therefore, there may be undescribed species, especially given the geographical location of TARP on the edge of the Coral Triangle.

The present study is the first investigation into the stoloniferous octocorals in and around the TARP area, off the coast of Kota Kinabalu, Sabah, Malaysia, and aims to improve on our understanding of the phylogenetic relationships of stoloniferous octocorals within the Octocorallia radiation. Based on our findings, we formally describe two new monotypic genera within Arulidae and three new species within the genus *Phenganax* Alderslade & McFadden, 2011 within Clavulariidae.

## Materials and methods

### Specimen collection

**Figure 1.**
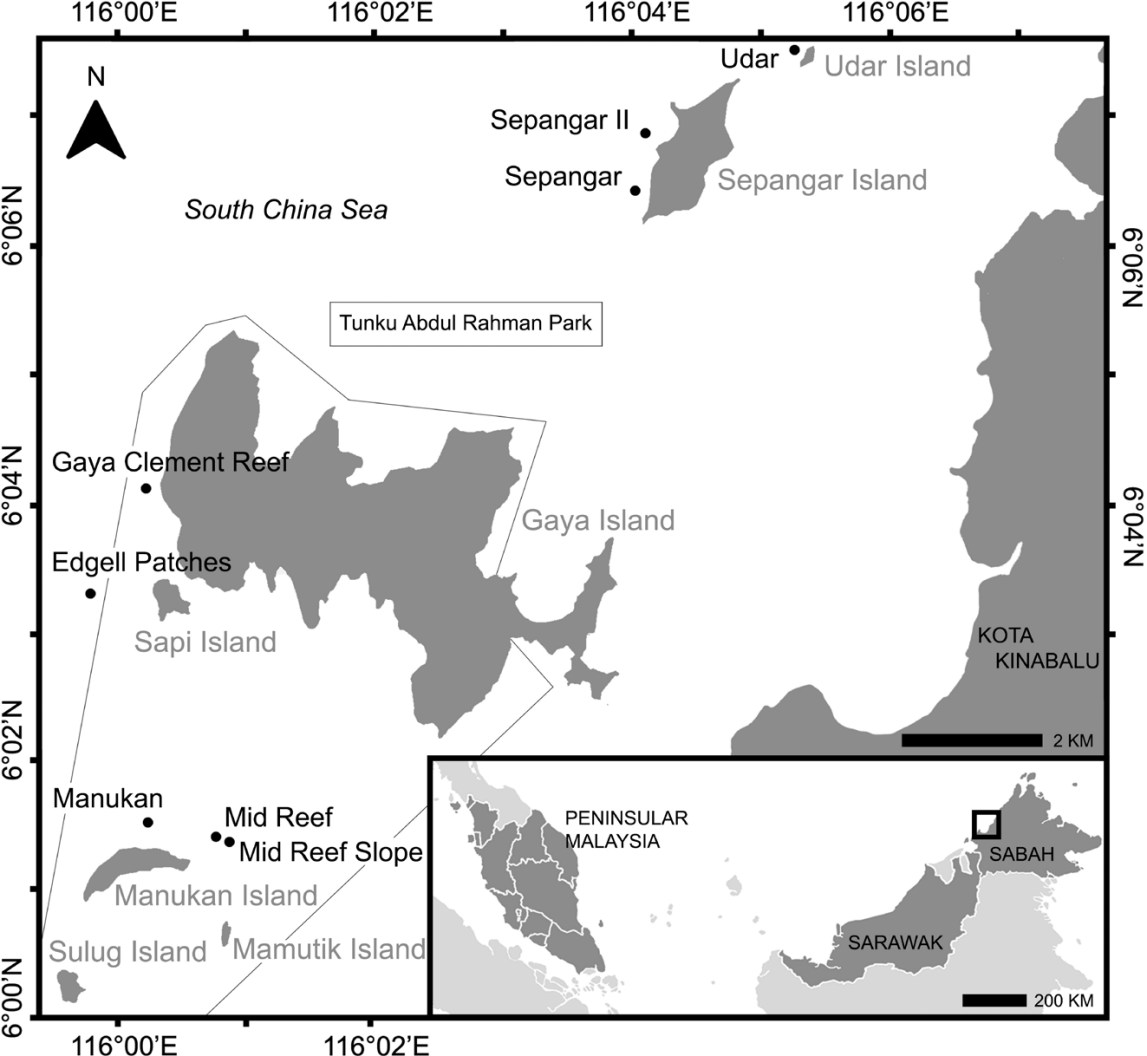
Map of eight sampling locations where stoloniferous octocorals were collected in this study, around Udar and Sepangar Islands and within Tunku Abdul Rahman Park (including Gaya, Sapi, Manukan, Mamutik, and Sulug Islands), Kota Kinabalu, Sabah, Malaysia.

A total of 25 stoloniferous octocoral specimens (Table 1) was collected from eight locations (Figure 1) in and around TARP, off the coast of Kota Kinabalu, Sabah, Malaysia, between 20 and 27 March 2018. Specimens were collected at depths of 4–18 m by means of SCUBA and all voucher material was preserved in 70–80% ethanol and sub-samples in 95% ethanol. High-resolution in situ images were taken with an Olympus Tough TG-4 in an Olympus PT-056 underwater housing. Vouchers and type material have been deposited at the National Museum of Nature and Science (**NSMT**), Tokyo, Japan (all holotypes) and at the Borneo Marine Research Institute (**IPMB**), Universiti Malaysia Sabah (UMS), Sabah, Malaysia (all paratypes).

**Table 1.** Overview of stoloniferous octocoral specimens collected from off NW Sabah, used in this study; including GenBank accession numbers and locality. Key: catalogue number: NSMT = National Museum of Nature and Science, Tokyo, Japan; IPMB = Borneo Marine Research Institute, Sabah, Malaysia; n.a. = not available.

Family	Species	Catalogue/voucher number	Location	GPS (DMS)	GenBank accession numbers			
					28S rDNA	COI	mtMutS	ND6
Arulidae	*Bunga payung* gen. nov. et sp. nov.	IPMB-C 01.00017	Sepangar, E Sepangar Is.	06°03'38.66"N, 116°04'0.65"E	MN164539	n.a.	n.a.	MN164587
	*B. payung* gen. nov. et sp. nov. (holotype)	NSMT-Co 1679	Sepangar, E Sepangar Is.	06°03'38.66"N, 116°04'0.65"E	MN164540	MN164559	n.a.	MN164588
	*Laeta waheedae* gen. nov. et sp. nov.	IPMB-C 01.00018	Mid Reef, E Manukan Is.	05°58'35.8"N, 116°00'52.2"E	MN164542	MN164561	MN164583	MN164590
		IPMB-C 01.00019	Gaya Clement Reef, E Gaya Is.	06°01'24.26"N, 116°00'13.55"E		MN164562	MN164584	MN164591
	*L. waheedae* gen. nov. et sp. nov. (holotype)	NSMT-Co 1680	Udar, W Udar is.	06°4'49.81"N, 116°5'13.16"E	MN164541	MN164560	MN164582	MN164589
Clavulariidae	Clavulariidae sp.	NSMT-Co 1686	Edgell Patches, W Sapi Is.	06°00'38.7"N, 115°59'22.2"E	MN164543	MN164563	MN164580	n.a.
		IPMB-C 01.00016	Gaya Clement Reef, W Gaya Is.	06°01'24.26"N, 116°00'13.55"E	MN164544	MN164564	MN164581	n.a.
	*Phenganax marumi* sp. nov.	IPMB-C 01.00001	Edgell Patches, W Sapi Is.	06°00'38.7"N, 115°59'22.2"E	MN164545	MN164570	n.a.	MN164595
		IPMB-C 01.00002	Mid Reef Slope, E Manukan Is.	05°58'38.08"N, 116°00'52.82"E	MN164546	MN164571	n.a.	MN164596
		IPMB-C 01.00003	Mid Reef Slope, E Manukan Is.	05°58'38.08"N, 116°00'52.82"E	n.a.	MN164572	n.a.	MN164597
		IPMB-C 01.00004	Edgell Patches, W Sapi Is.	06°00'38.7"N, 115°59'22.2"E	MN164547	MN164573	n.a.	MN164598
		IPMB-C 01.00005	Edgell Patches, W Sapi Is.	06°00'38.7"N, 115°59'22.2"E	MN164548	MN164574	n.a.	MN164599
		IPMB-C 01.00006	Gaya Clement Reef, W Gaya Is.	06°01'24.26"N, 116°00'13.55"E	MN164549	MN164575	n.a.	MN164600
		IPMB-C 01.00007	Gaya Clement Reef, W Gaya Is.	06°01'24.26"N, 116°00'13.55"E	n.a.	MN164576	n.a.	MN164601
		IPMB-C 01.00008	Manukan, N Manukan Is.	05°58'46.1"N, 116°00'10.6"E	MN164550	MN164577	n.a.	MN164602
	*P. marumi* sp. nov. (holotype)	NSMT-Co 1683	Manukan, N Manukan Is.	05°58'46.1"N, 116°00'10.6"E	MN164551	n.a.	n.a.	MN164603
	*P. marumi* sp. nov.	IPMB-C 01.00009	Manukan, N Manukan Is.	05°58'46.1"N, 116°00'10.6"E	MN164552	MN164578	n.a.	MN164604
		IPMB-C 01.00010	Manukan, N Manukan Is.	05°58'46.1"N, 116°00'10.6"E	MN164553	MN164579	n.a.	MN164605
	*Phenganax subtilis* sp. nov. (holotype)	NSMT-Co 1684	Sepangar, W Sepangar Is.	06°03'38.66"N, 116°04'0.65"E	MN164554	MN164566	MN164586	n.a.
	*P. subtilis* sp. nov.	IPMB-C 01.00011	Sepangar II, W Sepangar Is.	06°04'7.38"N, 116°04'6.76"E	MN164555	MN164567	n.a.	n.a.
		IPMB-C 01.00013	Manukan, N Manukan Is.	05°58'46.1"N, 116°00'10.6"E	n.a.	MN164568	n.a.	n.a.
		IPMB-C 01.00012	Manukan, N Manukan Is.	05°58'46.1"N, 116°00'10.6"E	n.a.	MN164569	n.a.	n.a.
	*Phenganax stokvisi* sp. nov. (holotype)	NSMT-Co 1685	Mid Reef, E Manukan Is.	05°58'35.8"N, 116°00'52.2"E	MN164556	n.a.	n.a.	MN164592
	*P. stokvisi* sp. nov.	IPMB-C 01.00015	Mid Reef, E Manukan Is.	05°58'35.8"N, 116°00'52.2"E	MN164557	n.a.	n.a.	MN164593
		IPMB-C 01.00014	Mid Reef, E Manukan Is.	05°58'35.8"N, 116°00'52.2"E	MN164558	MN164565	MN164585	MN164594

### Morphological examinations

Sclerites were isolated by dissolving different parts of the specimens (polyp tentacle, calyx, entire polyp and stolon) in 4% hypochlorite (household bleach). Rinsing and visualisation of sclerites followed the same protocol as described in Lau et al. (2018, 2019). Additionally, sclerites from specimens that needed more detailed examination were mounted on scanning electron microscope stubs and coated with Pd/Au for imaging on a JEOL JSM6490LV scanning electron microscope (SEM) operated at high vacuum at 15 kV.

### DNA extraction, amplification, and sequencing

DNA was extracted from polyps using a DNeasy Blood and Tissue kit (Qiagen, Tokyo). PCR amplification and sequencing of three mitochondrial markers, cytochrome c oxidase subunit I (COI), mitochondrial mutS-like protein (mtMutS), and the ND6 subunit (ND6), and one nuclear ribosomal marker, 28S ribosomal DNA (28S rDNA) followed the protocols in Lau et al. (2019). The amplified products were visualised by 1% agarose gel electrophoresis. PCR products were treated with Exonuclease I and Alkaline Phosphate (Shrimp) and sent for bidirectional sequencing on an ABI 3730XL (Fasmac, Kanagawa, Japan). Sequences were assembled and edited using Geneious R11 (Kearse et al. 2012) and BioEdit (Hall 1999). COI, mtMutS, and ND6 were checked for introns, exons, and stop-codons in AliView (Larsson 2014).

### Molecular phylogenetic analyses

Multiple sequence alignments were performed using MAFFT 7 (Katoh and Standley 2013) and coding markers were aligned using MACSE (Ranwez et al. 2011) under default parameters. To determine the phylogenetic position of the collected specimens, consensus sequences for markers 28S rDNA, COI and mtMutS were aligned to a reference dataset of 121 octocoral genera (n = 127 sequences), including sequences of *Cornularia pabloi* McFadden & van Ofwegen, 2012 and *Cornularia cornucopiae* (Pallas, 1766) as outgroup (total n = 134 sequences). Alignments of 893 bp for 28S rDNA, 717 bp for COI and 923 bp for mtMutS were separately run in Maximum Likelihood (ML) analyses (Suppl. material 1: Figures S1–S3), which were highly congruent with the phylogenetic construction generated with the concatenated three-marker dataset (2533 bp).

A separate phylogenetic analysis was made using a concatenated four-marker dataset to further investigate the phylogenetic position of collected clavulariid and arulid specimens. The four separate markers (28S rDNA, 804 bp; COI, 717 bp; mtMutS, 734 bp; ND6, 441 bp) were also run in ML analyses to check for congruency (Suppl. material 1: Figures S4–S7). The concatenated markers resulted in a 2696 bp dataset with a total of nine reference taxa, including *Hanabira yukibana* Lau, Stokvis, Imahara & Reimer, 2019 as outgroup (total n = 33).

Alignments of the separate markers were all concatenated using SequenceMatrix 1.8 (Gaurav et al. 2011). ML analyses were run with RAX-ML 8 (Stamatakis 2014) using the GTRCAT model. The best ML tree was calculated using the –D parameter. A multi-parametric bootstrap search was performed, which automatically stopped based on the extended majority rule criterion. The Bayesian inference was performed with ExaBayes 1.5 (Aberer et al. 2014) using the GTR substitution model. Four independent runs were run for 10,000,000 generations during which convergence (with a standard deviation of split frequencies <2%) had been reached. Bootstrap supports and posterior probabilities were depicted on the branches of the best ML tree using P4 (Foster 2004). The resulting tree was visualized in FigTree 1.4.2 (Rambout 2014). Additionally, average distance estimations within species and within genera were computed using MEGA X (Kumar et al. 2018) by analysing pairwise measures of genetic distances (uncorrected *P*) among sequences (Suppl. material 1: Tables S1–S6).

## Systematic account

### Class Anthozoa

#### Subclass Octocorallia Ehrenberg, 1831

##### Order Alcyonacea Lamouroux, 1812

###### Family Arulidae McFadden & Ofwegen, 2012

####### Genus Bunga gen. nov.

Taxon classificationAnimaliaAlcyonaceaArulidae

7B9D9009A0D150828F13299B08B1C31F

http://zoobank.org/BFF20AFD-A854-48EB-9F6A-EB3AC2066A95

######## Type species.

*Bunga payung* sp. nov., by original designation and monotypy.

######## Diagnosis.

Colony with polyps connected through thin stolons, which are cylindrical in cross-section and loosely attached to hard substrate. Anthocodiae retract into clavate calyces, which do not retract into the stolon. Oral disk expanded to circular membrane, as is characteristic of arulids. Oral disk with eight shallow furrows running from intertentacular margin to mouth of polyp, dividing membrane into eight lobes. Distal two-thirds of tentacles extend from fused margins of oral membrane. Sclerites of anthocodiae are rods. Sclerites of calyx are table-radiates. Sclerites of stolon are fused table-radiates forming a sheet. Sclerites colourless. Zooxanthellate.

######## Remarks.

The main difference between type species *Bunga payung* gen. nov. et sp. nov., with *Arula* McFadden & Ofwegen, 2012 and *Hana*Lau et al., 2018 is found in both polyp morphology and sclerites; the outer margins of the oral membrane are much less pronounced than seen in *Arula* and *Hana*, as well as the lobes, as the furrows appear to be shallower. The 6-radiate sclerite type could not be found in any of the *Bunga* gen. nov. specimens, which are present in the polyp calyx of both *Arula* and *Hana*. *Bunga*, similar to *Hana*, has fused table-radiates that form a sheet in the stolon.

######## Etymology.

From the Malaysian and Indonesian word *bunga*, meaning flower; denoting the shape of the polyps, which resemble flowers. Gender: feminine.

####### Bunga payung sp. nov.

Taxon classificationAnimaliaAlcyonaceaArulidae

A7BFFA7C748755D79DECEC7906461195

http://zoobank.org/5DDA8DCE-2333-4C45-8FA4-77021E54A211

Figures 2a–c , 4

######## Material examined.

All specimens are from Sepangar, Sepangar Island, Kota Kinabalu, Sabah, Malaysia (06°03'38.66"N, 116°04'0.65"E), 20 March 2018 and collected by YW Lau. **Holotype**: NSMT-Co 1679, 9 m depth. **Paratype**: IPMB-C 01.00017, 10 m depth.

######## Description.

**Figure 2.**
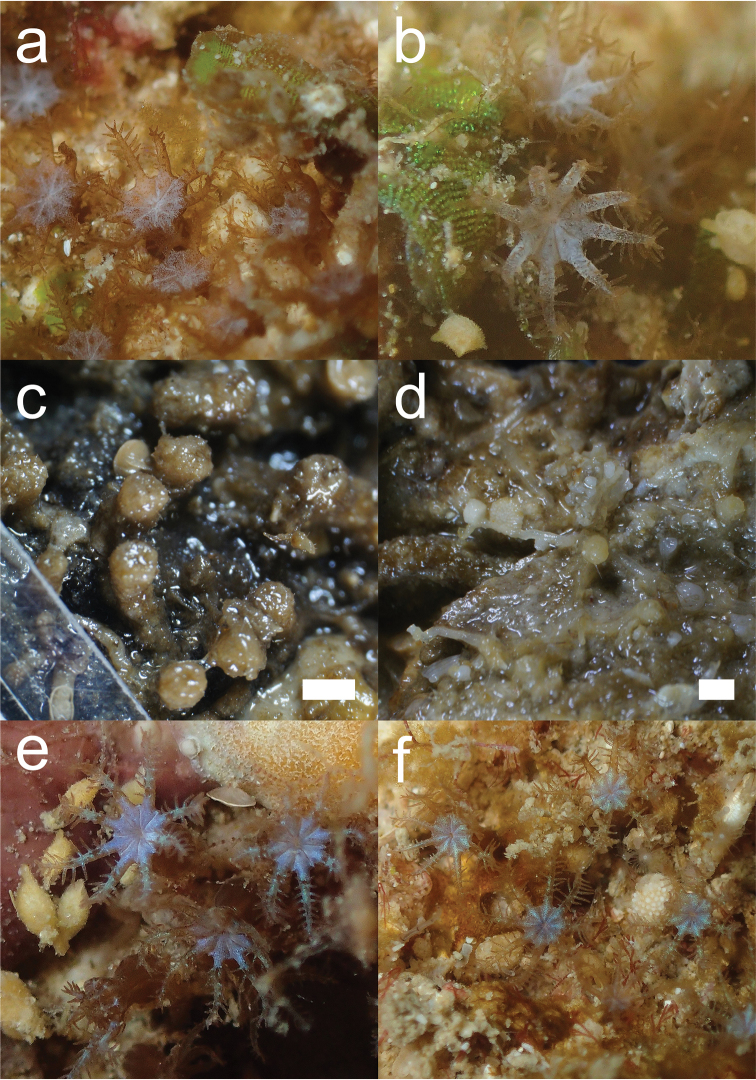
*Bunga payung* gen. nov. et sp. nov.: **a** In situ photograph, holotype (NSMT-Co 1679) **b** In situ photograph, paratype (IPMB-C 01.00017) **c** holotype in ethanol. *Laeta waheedae* gen. nov. et sp. nov. **d** holotype (NSMT-Co 1680) in ethanol **e** In situ photograph, holotype **f** In situ photograph, paratype (IPMB-C 01.00019). Scale bars: 1 mm.

Colony with numerous polyps (total ~70). Polyps connected through stolons attached to rock. Stolons are thin and rounded (circular in cross-section, ~0.3 mm in diameter) and polyps are spaced apart irregularly, either adjacent to one another or spaced apart up to ~5 mm. Expanded polyps are ~2.2–3.0 mm in width and retract fully into calyces of ~1 mm wide and up to ~2 mm in height. Calyces do not retract into the stolon. The oral disk of the polyps is expanded into a circular membrane by fusion of proximal regions of adjacent tentacles (Figure 2a), as is characteristic of arulids. The oral disk has eight shallow furrows that run from intertentacular margin to mouth of polyp, dividing the membrane into eight lobes. The distal two-thirds of the tentacles extend from fused margins of the oral membrane. Tentacles with 6–10 pairs of widely spaced pinnules are arranged in a single row on either side of rachis.

Anthocodial sclerites are smooth rods, with simple tubercles at distal margin ends, 0.1–0.15 mm long (Figure 4a). Calyces contain table-radiates that range 0.06–0.19 mm in length (Figure 4b–d). Sclerites of the stolon are fused table-radiates forming a flat sheet (Figure 4e).

Polyps are brown coloured in life with a whitish oral disk, but yellowish white when preserved in ethanol. Zooxanthellate.

######## Morphological variation.

The paratype is a colony consisting of ~10 polyps. Polyps of the paratype colony show variation in colouration; the whitish colour is not restricted to the oral disk but is also seen in the tentacles. This could be due to differences in sclerite density (Figure 2a), as described in Alderslade and McFadden (2007) and Lau et al. (2019), or it could be due to the position of zooxanthellae in the tissue.

######## Distribution.

Sepangar Island, Kota Kinabalu, Sabah, Malaysia.

######## Remarks.

Anthocodial rods are scarce in *Bunga payung* gen. nov. et sp. nov. The 6-radiate type of sclerite was not observed in this genus but is present in *Arula* and *Hana*, the two other genera in the family Arulidae.

######## Etymology.

From the Malaysian and Indonesian word *payung*, which means umbrella; denoting the shape of the oral disc of the polyps, which resemble the shape of an umbrella.

####### Genus Laeta gen. nov.

Taxon classificationAnimaliaAlcyonaceaArulidae

34C1E76EFA0854E1ADF3CA66CF0B00A9

http://zoobank.org/02B40F3A-AA78-49A5-ADA8-12F3374CDF21

######## Type species.

*Laeta waheedae* sp. nov., by original designation and monotypy.

######## Diagnosis.

Colony with polyps connected through stolons, which are ribbon-like. Oral disk with eight furrows, running from intertentacular margin to the mouth of the polyp, dividing the membrane into eight shallow lobes. Distal two-thirds of tentacles extend from fused margins of the oral membrane. Sclerites of anthocodiae are rods. Sclerites of calyx are table-radiates and three types of rods; (1) branched rods with high tuberculate processes, (2) club-like rods and (3) table-radiate-like rods. Sclerites of stolon are fused table-radiates, which form a sheet. Sclerites colourless. Zooxanthellate.

######## Remarks.

The main morphological difference between the type species, *Laeta waheedae* gen. nov. et sp. nov., and species of the genera *Arula*, *Hana*, and *Bunga* gen. nov. is found in the presence of all three types of rods in its calyces. The club-like rod with tubercles at one distal end of the rod is also seen in *Hana hanataba*Lau et al., 2018, however this type of sclerite was only found in the anthocodiae in *Laeta waheedae* and not in the calyx as in *Hana*. Similar to *Bunga*, *Laeta* does not have 6-radiates in the calyx. Similar to *Hana* and *Bunga*, *Laeta* has fused table-radiates in the stolon. Outer margins of the oral membrane are much less pronounced than in *Arula* and *Hana*, and are more similar to margins of the oral disk in *Bunga*.

######## Etymology.

From Latin *laeta*, meaning bright, charming, cheerful. Gender: feminine.

####### Laeta waheedae sp. nov.

Taxon classificationAnimaliaAlcyonaceaArulidae

289F7404F31D5F01A1C9F9599E3EF929

http://zoobank.org/761E7919-D311-4F0B-8B67-17E8ECDEC370

Figures 2d–f , 5

######## Material examined.

All specimens are from Kota Kinabalu, Sabah, Malaysia and collected by YW Lau. **Holotype**: NSMT-Co 1680, Udar, east of Udar Island (06°4'49.81"N, 116°5'13.16"E), 10 m depth. **Paratypes**: IPMB-C 01.00018, Mid Reef, east of Manukan Island, TARP (05°58'46.1"N, 116°00'10.6"E), 8 m depth. IPMB-C 01.00019, Gaya, Gaya Island, TARP (06°01'24.26"N, 116°00'13.55"E), 11 m depth.

######## Description.

The colony consists of ~50 polyps, which are connected through ribbon-like stolons (0.5–0.6 mm width). Polyps are spaced apart quite regularly (~3 mm). Anthocodiae retract fully into low oval to cylindrical calyces (~1 mm in width, ~0.5 mm in height), which do not retract into the stolon. Expanded polyps 3.5–4.0 mm in diameter. Oral membrane with eight shallow furrows running from intertentacular margin to mouth of polyp, dividing the membrane into eight shallow lobes. Distal two-thirds of tentacles extend from fused margins of the oral membrane. Tentacles with 6–8 pairs of widely spaced pinnules are arranged in a single row on either side of rachis.

**Figure 3.**
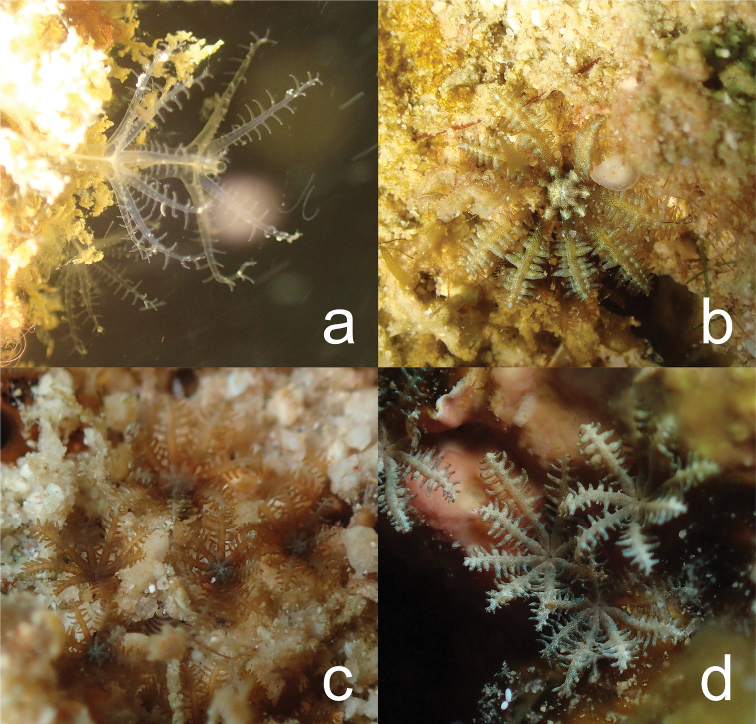
In situ photographs of clavulariids found in and around TARP, Kota Kinabalu, Sabah, Malaysia. **a**Clavulariidae sp., specimen NSMT-Co 1686 **b***Phenganax marumi* sp. nov., holotype (NSMT-Co 1683) **c***Phenganax subtilis* sp. nov., holotype (NSMT-Co 1684) **d***Phenganax stokvisi* sp. nov., holotype (NSMT-Co 1685).

Sclerites of anthocodiae are small smooth rods, 0.035–0.1 mm in length, with little ornamentation (Figure 5c). Sclerites of calyx are table-radiates, 0.06–0.10 mm (Figure 5d, e, g) and three types of rods: (1) rods branched with high tuberculate processes, 0.08–0.09 mm long (Figure 5f), (2) rods with tubercles at one distal end of the rod, giving it a club-like appearance, 0.1–0.14 mm long (Figure 5a), and (3) rods with table-shaped tubercles, 0.08–0.12 mm long (Figure 5b).

Oral membrane has a blue colour in life (yellowish white when preserved in ethanol). The tentacles are brown in colour, partially with a greenish shimmer. Zooxanthellate.

######## Morphological variation.

The paratype colony consists of ~30 polyps. Tentacles of paratypes show variation in pinnule pairs, partly having nine pairs of widely spaced pinnules arranged in a single row on either side of rachis.

######## Distribution.

Udar Island, Kota Kinabalu, Sabah, Malaysia. Gaya Island, TARP, Kota Kinabalu, Sabah, Malaysia.

######## Remarks.

Polyps of *Laeta waheedae* gen. nov. et sp. nov. have the same blue-purple colour in life as those of *Arula petunia* McFadden & Ofwegen, 2012, but there is a difference in the outer margins and lobes of the oral membrane; both are more pronounced in *Arula petunia*. The holotype colony was attached to sponge tissue, but this epibiotic relation is not obligate.

######## Etymology.

Named after Dr. Zarinah Waheed, for her dedication to coral reef research and her guidance during fieldwork in Sabah.

###### Family Clavulariidae Hickson, 1894

####### Genus Phenganax

Taxon classificationAnimaliaAlcyonaceaArulidae

Alderslade & McFadden, 2011

5B94F4202707519BA68EA0EAA5435705

######## Diagnosis.

(after Alderslade & McFadden 2011). Alcyonacea with erect polyps and stolons with encrusting, stoloniferous habit. Sclerites are absent. Zooxanthellate. Distribution tropical, Indo-Pacific. Type species: *Phenganax parrini* Alderslade & McFadden, 2011.

####### Phenganax marumi sp. nov.

Taxon classificationAnimaliaAlcyonaceaArulidae

27333CB5803E5D2685480AE7926D5905

http://zoobank.org/29410835-EB6E-4E9D-9361-CF60FE36ECFD

Figure 3b

######## Material examined.

All specimens are from Kota Kinabalu, Sabah, Malaysia and collected by YW Lau. **Holotype**: NSMT-Co 1683, Manukan, Manukan Island, TARP (05°58'46.1"N, 116°00'10.6"E), 13 m depth. **Paratypes**: IPMB-C 01.00001: Edgell Patches, west of Sapi Island (06°00'38.7"N, 115°59'22.2"E), 16 m depth. IPMB-C 01.00002: Mid Reef Slope, east of Manukan Island, TARP (05°58'38.08"N, 116°00'52.82"E), 13 m depth. IPMB-C 01.00003: Mid Reef Slope, east of Manukan Island, TARP (05°58'38.08"N, 116°00'52.82"E), 11 m depth. IPMB-C 01.00004: Edgell Patches, west of Sapi Island (06°00'38.7"N, 115°59'22.2"E), 19 m depth. IPMB-C 01.00005: Edgell Patches, west of Sapi Island (06°00'38.7"N, 115°59'22.2"E), 16 m depth. IPMB-C 01.00006: Gaya Clement Reef, west of Gaya Island, TARP (06°01'24.26"N, 116°00'13.55"E), 12 m depth. IPMB-C 01.00007: Gaya Clement Reef, west of Gaya Island, TARP (06°01'24.26"N, 116°00'13.55"E), 11 m depth. IPMB-C 01.00008: Manukan, north of Manukan Island, TARP (05°58'46.1"N, 116°00'10.6"E), 12 m depth. IPMB-C 01.00009: Manukan, Manukan Island, TARP (05°58'46.1"N, 116°00'10.6"E), 12 m depth. IPMB-C 01.00010: Manukan, Manukan Island, TARP (05°58'46.1"N, 116°00'10.6"E), 12 m depth.

######## Description.

Colony is attached to rock and sponge and consists of ~15 polyps. Stolons are consistent in width throughout the colony (~0.35 mm). Polyps are spaced apart irregularly (from 0.5 mm up to 6 mm) and retract fully into calyces; calyces do not retract into stolon and are approximately 1 mm in width and 2 mm in height. Expanded polyps 4.0–5.5 mm in width. The pinnules are arranged on either side of the rachis in pairs of 12–15, sometimes spaced apart irregularly. The pinnules give a swollen appearance and are mostly conical shaped, otherwise diamond-shaped. The colony is sclerite-free. Polyps are brownish yellow in colour, with a bright yellowish white oral disk in life (whitish yellow when preserved in ethanol). Tentacles have a greenish colour interrupted with brown specks. Zooxanthellate.

######## Morphological variation.

Paratypes IPMB-C 01.00003 and IPMB-C 01.00007 show variation in the colouration of the polyps, having a paler and pinkish appearance. There is also variation in the number of pinnule pairs (~8 pairs) and the space between the pinnules; pinnules are sometimes so densely packed there is no space between them. Like the holotype, all paratype colonies are small colonies consisting of ~20 polyps.

######## Distribution.

Gaya, Sapi and Manukan Islands, TARP, Kota Kinabalu, Sabah, Malaysia.

######## Remarks.

*Phenganax marumi* sp. nov. resembles *Phenganax parrini* Alderslade & McFadden, 2011 in polyp morphology, but differs in colour. The numerous and densely packed polyps described for a *P. parrini* colony (Alderslade and McFadden 2011) have not been observed in any of the 11 collected *P. marumi* colonies; instead the maximum number of polyps observed in one colony was approximately 35. However, the density of *P. parrini* could be caused by unnatural conditions (aquarium environment) in which it was kept for scientific research purposes (Alderslade and McFadden 2011). *Phenganax marumi* was found on exposed spots on the reef, not sheltered from strong current or light, as opposed to *P. parrini*, which was found in dimly lit, sheltered locations.

######## Etymology.

From the Japanese word *marumi*, which means round or rounded; denoting the plump shape of the polyp tentacles, which give the polyp an overall plump appearance.

####### Phenganax subtilis sp. nov.

Taxon classificationAnimaliaAlcyonaceaArulidae

7A41C7E03E8059E7B416FEFF6536434F

http://zoobank.org/D01BA048-BF0F-4285-B4D3-3D071B9D1653

Figure 3c

######## Material examined.

All specimens are from Kota Kinabalu, Sabah, Malaysia and collected by YW Lau. **Holotype**: NSMT-Co 1684, Sepangar, west of Sepangar Island (06°03'38.66"N, 116°04'0.65"E), 7 m depth. **Paratypes**: IPMB-C 01.00011: Sepangar II, west of Sepangar Island (06°04'7.38"N, 116°04'6.76"E), 7 m depth. IPMB-C 01.00012: Manukan, north of Manukan Island, TARP (05°58'46.1"N, 116°00'10.6"E), 12 m depth. IPMB-C 01.00013: Manukan, north of Manukan Island, TARP (05°58'46.1"N, 116°00'10.6"E), 12 m depth.

######## Description.

The colony consists of ~50 polyps, which are connected through thin rounded stolons with a width of approximately 0.13–0.2 mm, growing over coral rubble. Polyps are partly clustered and spaced apart irregularly (1.0–3.0 mm). Polyps retract into calyces (0.65–0.77 mm width) that are barrel shaped. Expanded polyps are 3.0–3.5 mm in width and have tentacles with pinnules that are widely spaced and arranged in pairs of 12 on either side of the rachis. No sclerites were found in any of the specimens. Polyps are brown coloured in life and whitish yellow when preserved in ethanol. Zooxanthellate.

######## Morphological variation.

Paratypes show variation in the number of pinnules on either side of the rachis (10–13 pairs).

######## Distribution.

Sepangar Island, Kota Kinabalu, Sabah, Malaysia. Manukan Island, TARP, Kota Kinabalu, Sabah, Malaysia.

######## Remarks.

*Phenganax subtilis* sp. nov. differs from *P. parrini* and *P. marumi* sp. nov. mainly in the shape of the pinnules. *Phenganax subtilis* does not have the plump, diamond-shaped pinnules as seen in *P. marumi* and *P. parrini*. Additionally, the colour of *P. subtilis* is different from *P. parrini* and *P. marumi*; brown instead of the more characteristic white-grey.

######## Etymology.

From Latin *subtilis*, meaning simple, subtle, plain; denoting the subtleness of the polyps blending into the reef background. Gender: masculine.

####### Phenganax stokvisi sp. nov.

Taxon classificationAnimaliaAlcyonaceaArulidae

DD32586E0B2E5DD7BE5BF6DB1821F2E1

http://zoobank.org/5871284F-05F8-469D-AED1-0BE797C0FEB2

Figure 3d

######## Material examined.

All specimens are from dive location Mid Reef, east of Manukan Island (05°58'35.8"N, 116°00'52.2"E) TARP, Kota Kinabalu, Sabah, Malaysia and collected by YW Lau. **Holotype**: NSMT-Co 1685, 5 m depth. **Paratypes**: IPMB-C 01.00015: 4 m depth. IPMB-C 01.00014 4 m depth.

######## Description.

The colony consists of ~100 polyps which are densely packed and are connected through flattened ribbon-like stolons with irregular width (~0.21–0.55 mm). The colony is fragmented into two pieces with the rock it was attached to. Expanded polyps are approximately 3.0 mm in diameter and have tentacles with pinnules arranged in pairs of ten on either side of the rachis. Polyps are clustered closely together (adjacent to one another) and partly spaced apart (~1.0 mm in between clustered polyps). Polyps retract into calyces (~1.0 mm width) that are barrel shaped. No sclerites were found. Polyps are whitish grey coloured in life (whitish yellow when preserved in ethanol). Zooxanthellate.

######## Distribution.

Manukan Island, TARP, Kota Kinabalu, Sabah, Malaysia.

######## Remarks.

*Phenganax stokvisi* sp. nov. shows similarity to the densely packed *P. parrini*, polyps being in the same size range (2.5–3.0 mm width in *P. parrini*), only showing difference in the number of pinnule pairs and colour of the polyps (possibly due to zooxanthellae).

######## Etymology.

Named after Frank Robert Stokvis, whose passion for octocoral research has never changed since his first study on this topic.

####### Clavulariidae

Taxon classificationAnimaliaAlcyonaceaArulidae

sp.

4F253F95E71D5A93952393F39A2E08B4

Figure 3a

######## Material examined.

All specimens are from Kota Kinabalu, Sabah, Malaysia. NSMT-Co 1686, Edgell Patches, west of Sapi Island (06°00'38.7"N, 115°59'22.2"E), 18 m depth, coll. YW Lau. IPMB-C 01.00016, Gaya Clement Reef, west of Gaya Island, TARP (06°01'24.26"N, 116°00'13.55"E), 11 m depth, coll. YW Lau.

######## Description.

Colonies with 20–30 polyps are connected through flattened stolons, which have a varying width of 0.5–1 mm. Colonies can be loosely attached to sponge or rocky substrates, such as coral rubble. Polyps are transparent and clustered in groups, connected by stolons with lengths up to 4–5 mm. Expanded polyps were ~6.0–7.0 mm in width when alive, with the pharynx visible in all polyps. Polyps retract fully into the calyx, which is cylinder-shaped (~1.3 mm width and up to 1.5 mm tall) and do not retract fully into the stolon. The tentacles have approximately 11 pairs of pinnules, which are widely spaced apart. No sclerites were found in the specimens. Polyps are whitish translucent when alive (yellowish white when preserved in ethanol). Azooxanthellate.

######## Distribution.

West of Sapi and Gaya Islands, TARP, Kota Kinabalu, Sabah, Malaysia.

######## Remarks.

This material, henceforth Clavulariidae sp., can be identiﬁed to the family level Clavulariidae Hickson, 1894 by its initial morphological resemblance to the type species *Azoriella bayeri* Lopez-Gonzalez & Gili, 2001 and *Cervera atlantica* Lopez-Gonzalez et al., 1995 in having similar whitish translucent polyps, although, the polyps of *C. atlantica* are translucently orange. However, more diagnostic morphological features and more specimens are necessary before a genus- and species-level distinction can be made.

The main difference between Clavulariidae sp. and *A. bayeri* can be found in the absence of sclerites in Clavulariidae sp. Additionally, both type species *C. atlantica* and *A. bayeri* have polyps that are smaller than in Clavulariidae sp.; *C. atlantica*, ~5.1 mm width, *A. bayeri*, ~3.6 mm width, and Clavulariidae sp., ~6.0–7.0 mm width. As well, *C. atlantica* and *A. bayeri* have more pinnules on either side of the tentacles than seen in Clavularidae sp.; both *C. atlantica* and *A. bayeri* have 12–14 pinnules and Clavulariidae sp. has tentacles with 11 pairs.

**Figure 4.**
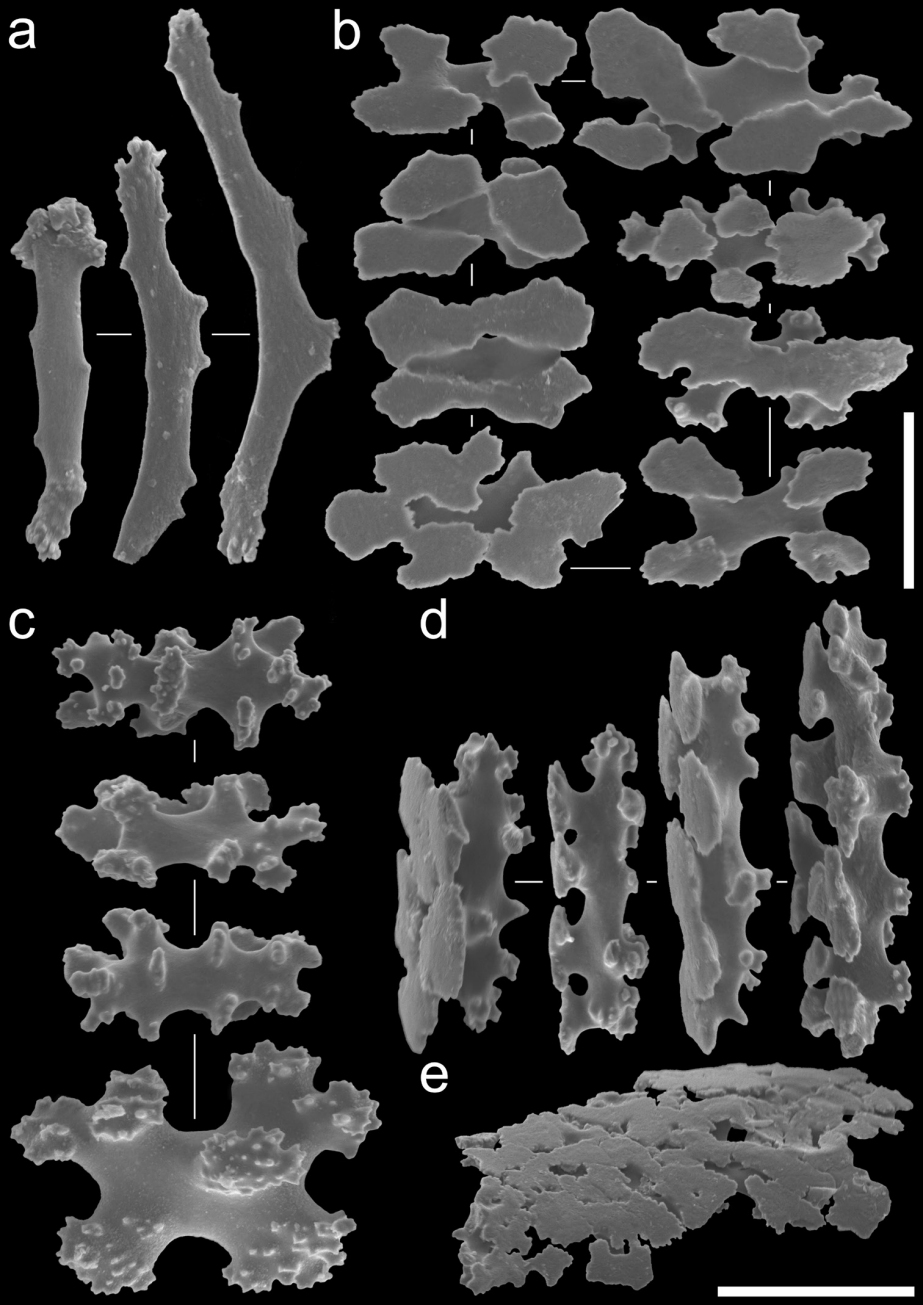
Sclerite types observed in *Bunga payung* gen. nov. et sp. nov., NSMT-Co 1679, holotype: **a** Anthocodial rods **b** table-radiates of calyx, top view **c** table-radiates of calyx, bottom view **d** table-radiates of calyx, lateral view **e** fragment of fused table-radiates of the stolon. Scale bars: 0.05 mm (**a–d**); 0.1 mm (**e**).

**Figure 5.**
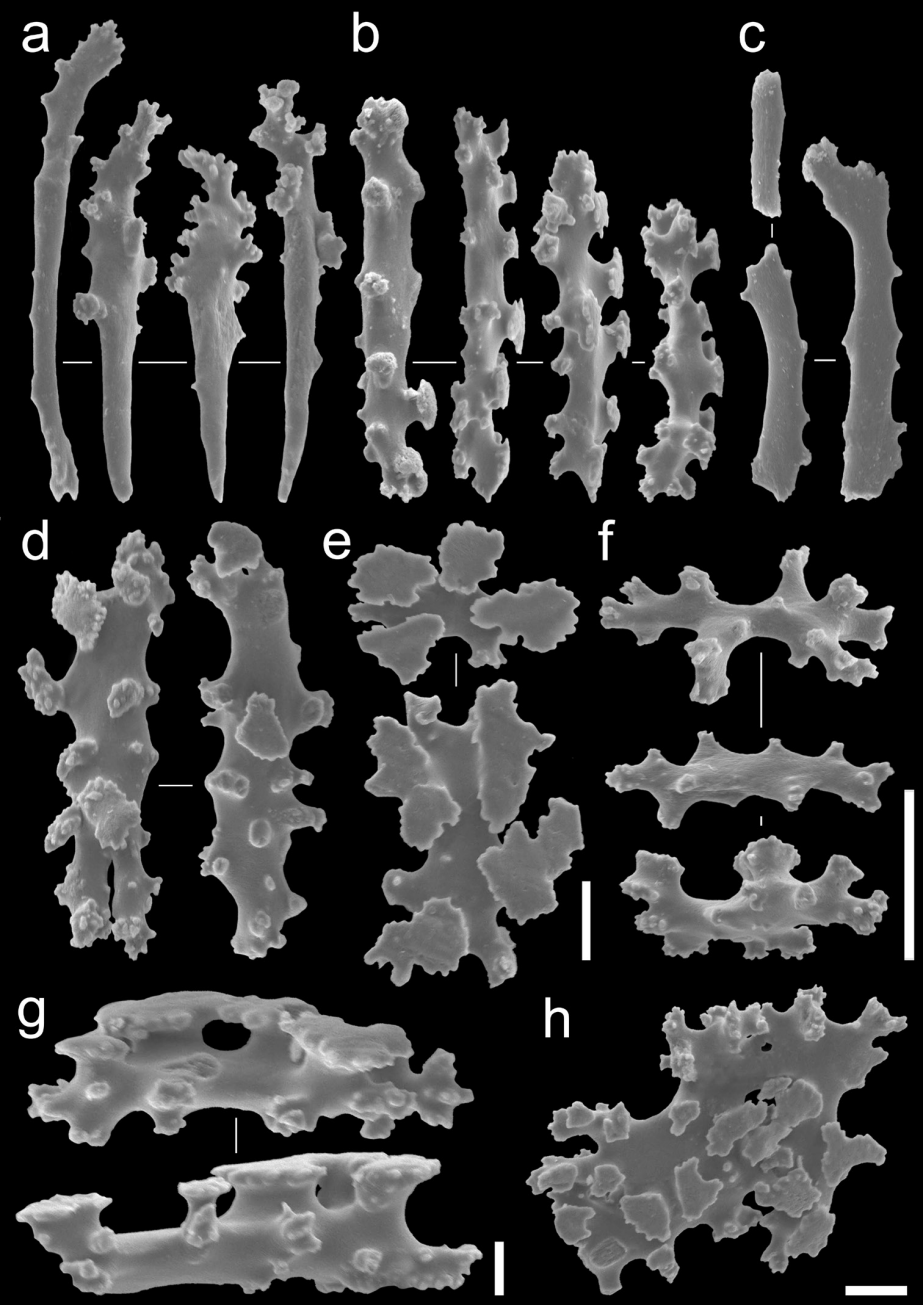
Sclerite types observed in *Laeta waheedae* gen. nov. et sp. nov., NSMT-Co 1680, holotype: **a** rods of calyx **b** rods of calyx **c** anthocodial rods **d** table-radiates of calyx **e** table-radiates of calyx, top view **f** rods of calyx **g** table-radiates of calyx, lateral view **h** fragment of fused table-radiates of the stolon. Scale bars 0.05 mm (**a–c, f**); 0.02 mm (**d–e**); 0.01 mm (**g**); 0.02 mm (**h**).

## Molecular phylogenetic analyses

**Figure 6.**
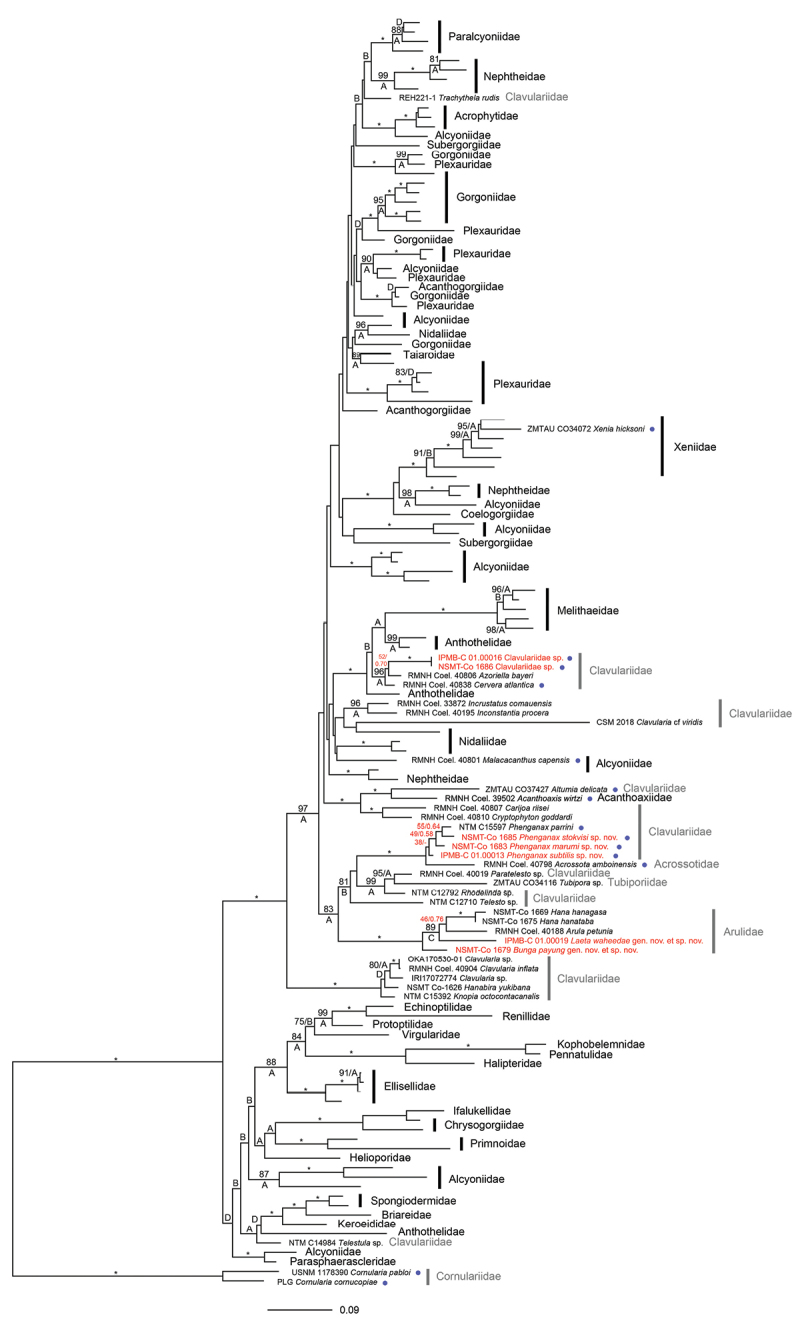
Phylogenetic relationships among 121 octocoral genera, including seven specimens (highlighted red) collected in and around TARP, Kota Kinabalu, Sabah, Malaysia, using the combined 28S rDNA+COI+mtMutS dataset (total n = 127). The best Maximum Likelihood tree is shown, with values at branches representing bootstrap probabilities (shown when > 70%; top/left) and Bayesian posterior probabilities (shown when > 0.80; bottom/right; A = 1.00, B = 0.95–0.99, C = 0.90–0.94, D = 0.80–0.89). Key: * represents 100%/1.00 for both analyses. Important values concerning target specimens are red. Non-stoloniferous families are shown with family classification only and stoloniferous families are highlighted in grey. Sclerite-free species are indicated with a blue dot. *Cornularia* spp. were used as outgroup.

**Figure 7.**
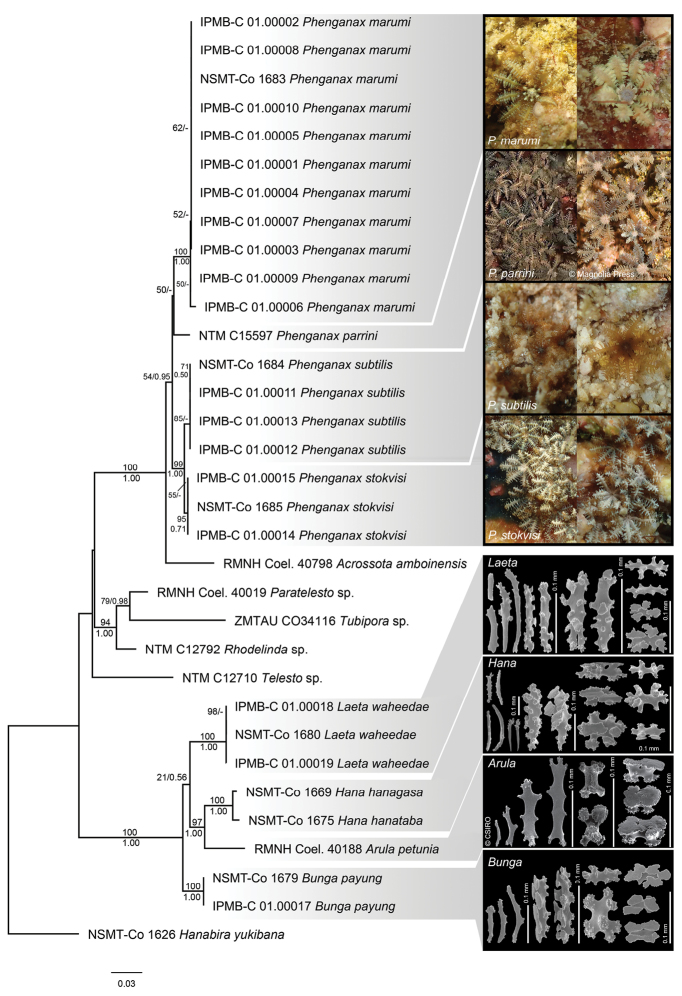
Combined 28S rDNA+COI+mtMutS+ND6 phylogenetic reconstruction for four *Phenganax* species and four arulid genera (*Hana*, *Arula*, *Bunga* gen. nov., and *Laeta* gen. nov.) collected in and around TARP, Kota Kinabalu, Sabah, Malaysia; *Hanabira yukibana* was used as outgroup (total n = 33). The best Maximum Likelihood tree is shown, with values at branches representing bootstrap probabilities (shown when > 50%; top/left) and Bayesian posterior probabilities (shown when > 0.50; bottom/right). Photograph credit: in situ images NTM C15597 *Phenganax parrini*, by Daniel Knop (modified from Alderslade and McFadden 2011, reproduced with permission from copyright holder). Sclerite images RMNH Coel. 40188 *Arula petunia* (modified from McFadden and Van Ofwegen 2012, reproduced with permission from CSIRO Publishing).

This study has produced 67 sequences, which were added to the public database GenBank; the 51 clavulariid, and 16 arulid sequences had no previous barcodes. The phylogenies resulting from the ML analyses of the separate markers (Suppl. material 1: Figures S1–S7) were highly congruent with those from the concatenated alignments for both the three- and four-marker datasets (Figures 6–7). ML and Bayesian analyses of the concatenated datasets yielded almost identical tree topologies.

### Arulidae from Sabah, Malaysia

Sequences of arulids *Bunga payung* gen. nov. et sp. nov. and *Laeta waheedae* gen. nov. et sp. nov., collected from the north of TARP (Udar, Gaya and Sepangar Islands) formed a completely-supported clade with the arulid genera *Arula* and *Hana*; 100%/1.00 for both the three- and four-marker datasets (Figures 6, 7, respectively). In both phylogenies, *Bunga* was sister to the remaining clade. However, it remains unresolved how *Laeta waheedae*, *Arula*, and *Hana* are related to one another in the four-marker phylogeny reconstruction (21%/0.56). Nonetheless, all genera in the clade have high support values.

Genetic distances (uncorrected *P*, expressed as percentage) between specimens of *Bunga* gen. nov., *Laeta* gen. nov., *Arula*, and *Hana* (between 2.6–4.6% for COI, 5.4–6.1% for mtMutS) indicated that the levels of pairwise differences were well above those observed among congeneric species (McFadden et al. 2011), Suppl. material 1: Table S1.

Morphological data supported the molecular data and justified the description of the two new genera *Bunga* and *Laeta*. There were distinct differences in sclerite features between *Bunga*, *Laeta*, *Arula*, and *Hana*, involving the presence and absence of sclerite types in the calyx; three types of rods (branched with high tuberculate processes, club-like rods and table-radiate-like rods), which were only observed in *Laeta* but not in the other sister genera, and the absence of 6-radiates in *Bunga*, which were present in all sister genera. There was also a difference in sclerites of the stolon; *Arula* is the only genus without fused table-radiates, but instead has table-radiates in the calyx. The polyp morphology did not show many obvious differences, although two of them could be observed in the outer margins of the characteristic fused oral membrane; both *Bunga* and *Laeta* had polyps with oral discs that were less pronounced at their outer margins than those of other genera, having a smooth transition from oral disc to the connecting tentacles, while the furrows dividing the oral disc into eight lobes were shallower in both of these genera (Figure 2). The combination of molecular and morphological information supports the erection of two new genera within the Arulidae.

### Clavulariidae from Sabah, Malaysia

Sequences of 18 clavulariid specimens grouped together with *Acrossota amboinensis* (Burchardt, 1902) and *Phenganax parrini* Alderslade & McFadden, 2011 in a well-supported clade in both the three-marker and four-marker analyses (Figures 6, 7, respectively), in which *Acrossota* was sister to the remaining clade (100%/1.00). However, there was disparity in the relationships within the remaining clade in the two phylogeny reconstructions and the relationships within the remaining clade were not clearly resolved. The four-marker phylogeny reconstruction, with more specimens, gave a higher resolution (Figure 7) and here, each species had high support values, although it remained unclear how *P. parrini* is related to the other three species (50%/-).

On the other hand, within-genus genetic distances for *Phenganax* were 0.57% for COI and 3.10% for mtMutS, percentages within the range of congeneric species commonly observed in octocorals (Suppl. material 1: Table S3). When comparing pairwise differences between the four species, the values were well above averages for conspecifics; the observed values were in between 0.98–4.09% for COI and between 1.60–4.07% for mtMutS (Suppl. material 1: Table S5), supporting the division into four species (McFadden et al. 2011).

Despite the absence of sclerites, which was characteristic for the entire clade, there were visible differences in morphological polyp features. The main differences were found in tentacle pinnules and colony growth (Figure 3b–d). *Phenganax marumi* sp. nov. was similar to *P. parrini* Alderslade & McFadden, 2011 in polyp morphology, although differing in colour and polyp size; expanded polyp sizes ranged 4.0–5.5 mm in *P. marumi* and 2.5–3.0 mm in *P. parrini*. Additionally, the numerous amounts and densely packed polyps described for a *P. parrini* colony (Alderslade and McFadden 2011) had not been observed in any of the 11 collected *P. marumi* colonies; instead the maximum number of polyps observed for one colony was approximately 30–40. However, the density of *P. parrini* could be due to unnatural conditions (aquarium environment) in which it was kept for scientific research (Alderslade and McFadden 2011).

*Phenganax marumi* sp. nov. was found on exposed spots on the reef, not sheltered from strong current or light, opposed to *P. parrini*, which was found in dimly lit, sheltered locations. *Phenganax subtilis* sp. nov. differs from *P. parrini* and *P. marumi* mainly in the shape of the pinnules; *P. subtilis* does not have the plump, diamond-shaped pinnules as seen in *P. marumi* and *P. parrini*. As well, the colour of *P. subtilis* is different from *P. parrini* and *P. marumi*, being brown. *Phenganax stokvisi* sp. nov. shows similarity to the densely packed *P. parrini*, with polyps being in the same size range (2.5–3.0 mm width), but showing differences in the number of pinnule pairs and colour of the polyps (possibly due to the presence of zooxanthellae).

Differences in morphology combined with genetic distances and high support values in the four-marker phylogeny reconstruction supported the erection of the three new sister species of *Phenganax parrini*; *P. marumi* sp. nov., *P. subtilis* sp. nov., and *P. stokvisi* sp. nov.

Clavulariidae sp. had a similar DNA profile to two genera within the Clavulariidae; sequences from the two specimens clustered in a well-supported clade (96%/1.00), together with the genera *Azoriella* (Lopez Gonzalez & Gili, 2001) and *Cervera* Lopez-Gonzalez et al., 1995, with *Cervera* as sister to *Azoriella* and Clavulariidae sp. (Figure 6). Genetic distances between the two specimens and *Azoriella* were 1.41% for COI and 5.09% for mtMutS, values within ranges amongst intergeneric octocoral species (Suppl. material 1: Table S2). However, with the current available molecular data it remains unresolved how Clavulariidae sp. and *Azoriella* are related to one another, as branching was weakly supported (52%/0/70).

## Discussion

From surveys at only eight different dive locations, approximately 13 hours of field work, all within approximately 60 km^2^ (TARP + area around Udar and Sepangar Islands), five species and two stoloniferan genera new to science were discovered. These results are not completely unexpected, as TARP is located on the outer edge of the Coral Triangle, where studies on stoloniferous octocorals are in their infancy. Of the seven families that are considered to belong to the subordinal group Stolonifera (Cordeiro et al. 2019), three have members that are confirmed to occur in the Indo-Pacific (Alderslade and McFadden 2007, 2011; Fabricius and Alderslade 2011): Acrossotidae Bourne, 1914, Tubiporidae Ehrenberg, 1828 and Clavulariidae Hickson, 1894. Of these, Clavulariidae is the only family that is not monogeneric or even monospecific, and is obviously the most speciose and common. The majority of recent studies on Indo-Pacific Clavulariidae have mainly focused on *Tubipora musica* Linnaeus, 1758, a reef-building alcyonarian species and *Tubipora musica*-sponge associations (Calcinai et al. 2013; Agustiadi and Luthfi 2017). Therefore, it is reasonable to assume that continued surveys in this area and within the Coral Triangle will result in further discoveries of undescribed stoloniferous octocorals. Such new descriptions could fill knowledge gaps in the overall systematics of Octocorallia, similar as recent discoveries of new small inconspicuous stoloniferan species have done (Alderslade and McFadden 2007, 2011; Benayahu et al. 2017; Lau et al. 2018, 2019).

### Arulids of Sabah

There are minor differences in polyp morphology when comparing *Bunga payung* gen. nov. et sp. nov., *Laeta waheedae* gen. nov. et sp. nov., *Arula petunia*, and *Hana* spp.; *Arula* and *Laeta* are similar in colour, having blue colorations in the oral disc and/or tentacles. *Hana* spp. are similar to *Arula petunia* in having a very pronounced oral disc with deep furrows dividing the oral disc into eight distinct lobes, as opposed to *Laeta* and *Bunga*, which both have an oral disc that fuses into the tentacle base and shallow furrows that divides the eight lobes.

It could therefore be proposed that instead of four different genera within Arulidae, *Arula*, *Hana*, *Bunga* gen. nov., and *Laeta* gen. nov. could be five distinct species of *Arula*. However, the erection of a new genus for each new species is justified by the combination of sclerite morphology and molecular information. The four genera of Arulidae are distinguished morphologically by differences in sclerite form and the presence or absence of certain sclerite types in different parts of the colony or being absent completely. The only two sclerite types that all four genera have in common are the rods present in the anthocodiae and the table-radiates in the calyx, which are characteristic to the family Arulidae. *Bunga* is the only genus that has only these sclerites types in the polyps; anthocodial rods and table-radiates in the calyx. *Arula*, *Hana*, and *Laeta* have an additional sclerite type in the calyx; *Arula* and *Hana* have 6-radiates. *Laeta* has three different ornamented rods in the calyx, which are absent in all other genera.

*Hana* and *Arula* are the two most similar genera in terms of morphology. However, *Arula* is the only genus which has separate table-radiate sclerites in the stolon, and it can also be distinguished from the other genera in having table-radiates that are smaller in size (up to 0.09 mm length) and by not having table-radiates which are elongated in shape; the other three genera all have stolons with fused table-radiates which form a sheet and all have larger table-radiates up to 0.10–0.19 mm length.

Molecular analyses of the concatenated four marker dataset (28s rDNA, COI, mtMutS, and ND6 sequences) resulted in similar phylogenetic positions for *Bunga payung* gen. nov. et sp. nov. and *Laeta waheedae* gen. nov. et sp. nov. when compared to our analyses of the concatenated three marker dataset (28s rDNA, COI, and mtMutS). It should be noted that the two analyses including more specimens for *Bunga* and *Laeta* placed the four genera in slightly different phylogenetic positions. In the three-marker dataset (Suppl. material 1: Figure S8), *Bunga* and *Laeta* formed a clade, although it was not very well supported (74/-). This clade was sister to *Hana* and *Arula*, which also formed a clade. In the four-marker dataset (Figure 7), *Bunga* was sister to all remaining clades. Nonetheless, branches of the four-marker dataset had better support values. Therefore, we again recommend the use of the extra gene region (ND6) next to the conventional DNA markers (COI, mtMutS, 28S rDNA) commonly used in octocoral phylogenetic analyses (Lau et al. 2018, 2019).

New discoveries and descriptions of stoloniferous octocorals outside of the Coral Triangle have shown surprising octocoral novelty, at the species, genus, and family levels (Alderslade and McFadden 2011; McFadden and Ofwegen 2012; Benayahu et al. 2017; Lau et al. 2018, 2019). Arulidae, the most recently described family of stoloniferous octocorals (McFadden and Ofwegen 2012), is a good example. Arulids have unique morphological features that had not been seen before in octocorals, including an expanded fused oral disc, and a new type of sclerite, table-radiates. The species *Bunga payung* gen. nov. et sp. nov. and *Laeta waheedae* gen. nov. et sp. nov. increase the total number of species within the family Arulidae to five. Moreover, they represent the first confirmed records of arulids in and around the shallow waters of the TARP area in Sabah, Malaysia. The discovery of these two monospecific taxa demonstrates that the recently erected family Arulidae is considerably much more diverse than was originally known. For example, there is photographic evidence of an arulid from the reefs of Bali, Indonesia (McFadden and Ofwegen 2012), and it is possible that other regions in or close to the Coral Triangle harbour unknown stoloniferan diversity.

The distinctive feature of the fused oral membrane seen in arulid polyps and its function is not yet fully understood. It has been proposed by Porter (1976) and Sebens (1979) that an increase in size of the capture surface area of polyps could be positively related to the amount of prey captured (Lasker 1981; Lewis 1982), as has also been suggested for scleractinian corals (Todd et al. 2004; Todd 2008). On the other hand, a large polyp size in scleractinians has been observed to be beneficial for the capture and consumption of large prey items (Alamura et al. 2009; Hoeksema and Waheed 2012; Mehrotra et al. 2014, 2016, 2019; Musco et al. 2018). A study on octocorals by Lasker (1981) showed that there were differences in the number of nematocysts, bigger polyps having relatively less nematocysts, which could influence prey capture. The expanded oral disc seen in arulids could perhaps be an adaptation for feeding and compensation for their relatively small polyp size and lower polyp number per colony, and it would be worthwhile to conduct a study of their nematocysts.

Another compelling hypothesis involves the possibility of these zooxanthellate taxa being heterotrophic, in which an increased light-gathering surface for photosynthesis of symbionts could explain the development of the expanded oral disc. Based on currently available information, the expanded oral disc does not appear to have evolved across the Octocorallia radiation, and is unique to the family Arulidae.

### Clavulariids of Sabah

Multiple factors made it initially difficult to establish with certainty if the currently described species were either congeners or possibly already described. The most important factor in this matter is the small number of morphological features that represent the new *Phenganax* species. The absence of sclerites makes it especially challenging to make confident judgements about evolutionary relationships between species within this genus. Additionally, the available historical literature on described stoloniferous species in the Indo- and northwestern Pacific is in a poor state. There are a number of *Clavularia* species listed by Alderslade and McFadden (2011) that could possibly belong to *Phenganax*, mainly due to the fact that these species also lack sclerites and the described distinguishing characters are alike: namely *Clavularia reptans* Hickson, 1894, *Clavularia reptans* sensu Thomson & Henderson (1906: 402), *Clavularia celebensis* Hickson, 1894, *Clavularia pregnans* Thomson & Henderson, 1906.

The main characteristic morphological difference between the *Phenganax* spp. described in this study and the above mentioned *Clavularia* spp. are the “numerous densely packed pinnules”, which are sometimes as many as 30 pinnules per row; all *Phenganax* spp. in the current study have six to 15 pinnules on either side of the tentacle rachis. Not all measurements (for example expanded polyp width) were included in the *Clavularia* spp. (Alderslade and McFadden 2011). *Clavularia reptans* was described as having polyps 7.0–10.0 mm width when expanded, whereas expanded polyp widths of *Phenganax* spp. are within the size range of 2.5–5.5 mm. Further information regarding the colouration of the tentacles and polyp body in combination with colony drawings make it doubtful that these *Clavularia* spp. belong to *Phenganax*. Additional arguments supporting the description of the *Phenganax* species in this study involve ecological aspects. The preferred habitat of *P. parrini* is dimly lit and in sheltered locations, below 10 m depth. The described *Phenganax* spp. in the current study, however, were found at exposed locations above 10 m depth.

Despite the fact that the relationships between the *Phenganax* spp., with emphasis on the position of *P. parrini*, seem to be unresolved, and all species lack sclerites to aid in their identification, the described species all had high support values and could be distinguished based on some features in colony growth form and polyp morphology, namely tentacle pinnules, the density of polyps, polyp size and spacing between polyps in a colony.

Similar to the new genera within Arulidae, the results for the *Phenganax* spp. also demonstrate that more specimens and the extra gene region (ND6) resulted in obtaining higher phylogenetic resolution and increased branch support values, and therefore should represent a more accurate reconstruction of evolutionary relationships. Future octocoral taxonomy will unquestionably build upon a combination of molecular and more traditional morphological techniques (Conti-Jerpe and Freshwater 2017); however, the lack of sclerites has important implications for future studies, as these skeletal parts are one of the major diagnostic features of the group. This emphasizes the need for improved molecular techniques, such as finding an optimum between concatenated marker datasets towards whole-genome mapping, combined with next-generation sequencing to allow more reliance on molecular information when morphological information is scarce.

The current and other recent works show that octocoral species without sclerites are not rarities; the absence of sclerites or other calciferous skeletal parts are known from within seven octocoral families (Acanthoaxiidae, Acrossotidae, Cornulariidae, Dendrobrachiidae, Alcyoniidae, Clavulariidae, Xeniidae) (Alderslade and McFadden 2007, 2011; López-González et al. 1995; Benayahu et al. 2017). Sclerites provide protection against predation, provide support and rigidity to soft colony parts, and are critical in octocoral classification (West 1997; Aharonovich and Benayahu 2012). Sclerites in different species differ in density as well as spatial organisation in the connective tissue, and thus they also differ in the degree to which they offer support and protection (Benayahu et al. 2017). It could be hypothesized that small size and inhabiting small cracks on reefs protects small stoloniferous species from exposure and has eliminated the need for sclerites in some of these species. Alternately, perhaps the absence of predation (e.g., by ovuliids or sea slugs) has made the possession of sclerites unnecessary. However, there is no obvious pattern to be found for this character in the octocoral phylogenetic radiation amongst its smallest members (Figure 6), and more work is needed to confirm this hypothesis. The absence of sclerites is an example of the gaps in our present knowledge about octocorals and indicate how much basic work in alpha taxonomy and documentation remains for this group.

Our results demonstrate that the TARP area, off the coast of Kota Kinabalu, in Sabah, Malaysia, harbours stoloniferous octocoral diversity that was previously unknown to science, which is obvious since it represents one of the first studies focusing on Stolonifera in this region. However, the newly described sclerite-free *Phenganax* spp. as well as Clavulariidae sp. are only small pieces of the puzzle in the needed thorough investigation of the polyphyletic family Clavulariidae (McFadden and Ofwegen 2012; Benayahu et al. 2017; Conti-Jerpe and Freshwater 2017; Lau et al. 2019), and an even smaller step towards a full understanding of the morphological and molecular distinctions amongst clades of Stolonifera and ultimately Octocorallia. Molecular data indicate that the entire Octocorallia classification needs re-structuring, but for now researchers still rely on the traditional morphological classification system (Fabricius and Alderslade 2001; Conti-Jerpe and Freshwater 2017), as evolutionary relationships are still far from being fully resolved (McFadden and Ofwegen 2012; Conti-Jerpe and Freshwater 2017). Integrative taxonomy combining not only morphology and molecular biology but also including ecology and biogeography data appears to be the best way to try to better understand the systematics of such a diverse and important group in marine benthic communities (Perez et al. 2016). To this end, marine parks should include in their goal not only the preservation of commercially important faunal diversity (primarily reef fish), but also of other reef organisms, and in particular, small and inconspicuous cryptic species, as it is these small reef inhabitants that make up the vast majority of the diversity of coral reef communities (Hoeksema 2017).

## Supplementary Material

XML Treatment for Bunga

XML Treatment for Bunga payung

XML Treatment for Laeta

XML Treatment for Laeta waheedae

XML Treatment for Phenganax

XML Treatment for Phenganax marumi

XML Treatment for Phenganax subtilis

XML Treatment for Phenganax stokvisi

XML Treatment for Clavulariidae

## Supplementary materials

Supplementary material 1 Figures S1–S8, Tables S1–S6 Data type: Adobe Acrobat Document (.pdf) https://binary.pensoft.net/file/328195This dataset is made available under the Open Database License (http://opendatacommons.org/licenses/odbl/1.0/). The Open Database License (ODbL) is a license agreement intended to allow users to freely share, modify, and use this Dataset while maintaining this same freedom for others, provided that the original source and author(s) are credited.Yee Wah Lau, James D. Reimer
