# Supplementary material for: A first phylogenetic study on stoloniferous octocorals off the coast of Kota Kinabalu, Sabah, Malaysia, with the description of two new genera and five new species
Source: Zookeys. 2019 Aug 26;872:127–58. doi: 10.3897/zookeys.872.36288 (PMC6718372; doi:10.3897/zookeys.872.36288)
Supplement: Supplementary material 1 [file zookeys-872-127-s001.pdf]

Supplementary material 1: Lau and Reimer (2019), ZooKeys

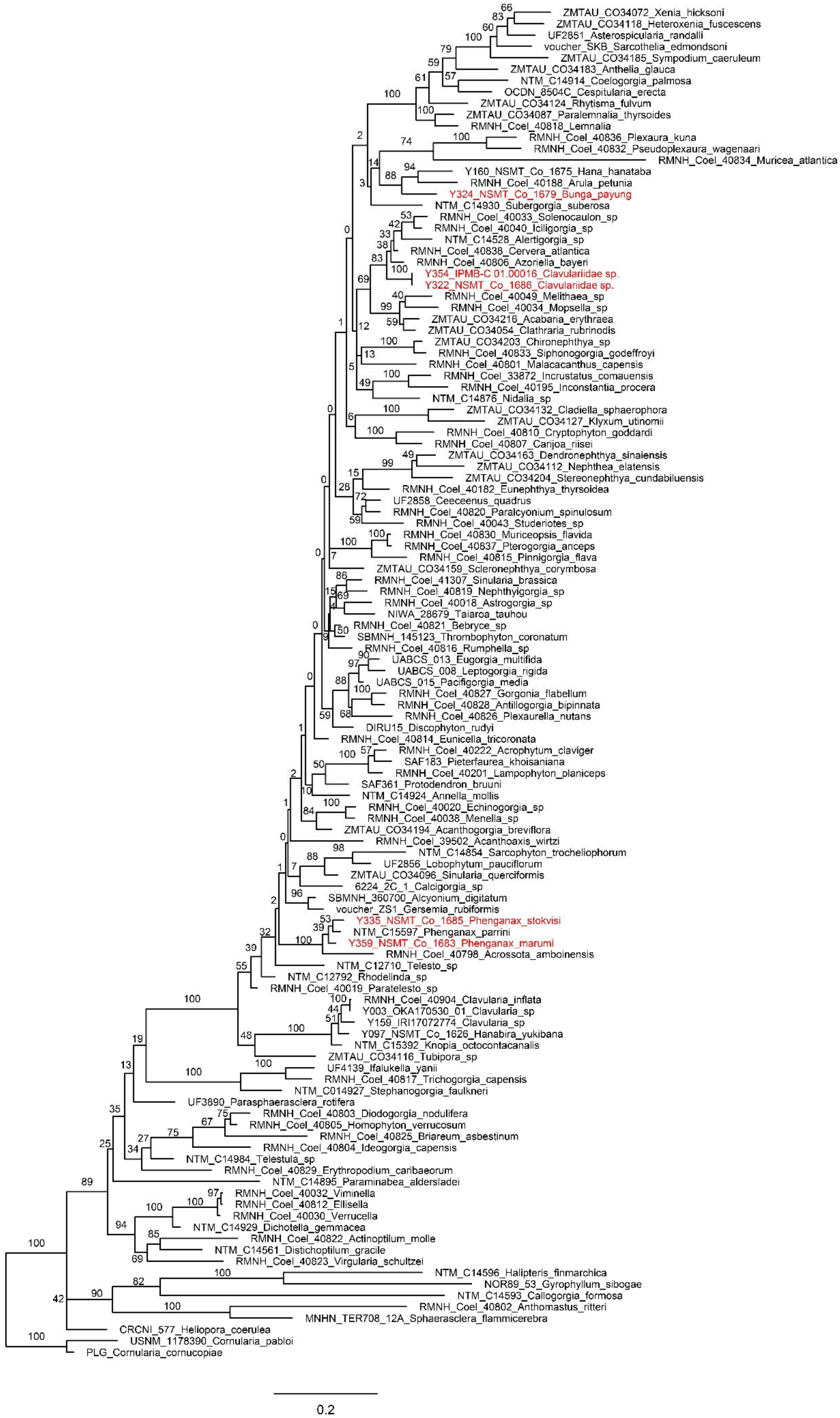

Figure S1. Maximum Likelihood phylogeny reconstruction of 28S rDNA gene region of stoloniferous octocorals (Clavulariidae sp., *Bunga payung* gen. nov. et sp. nov., *Phenganax marumi* sp. nov. and *Phenganax stokvisi* sp. nov.) from off the coast of NW Sabah (Malaysia), and octocoral references from 121 genera, including outgroup *Cornularia* spp.

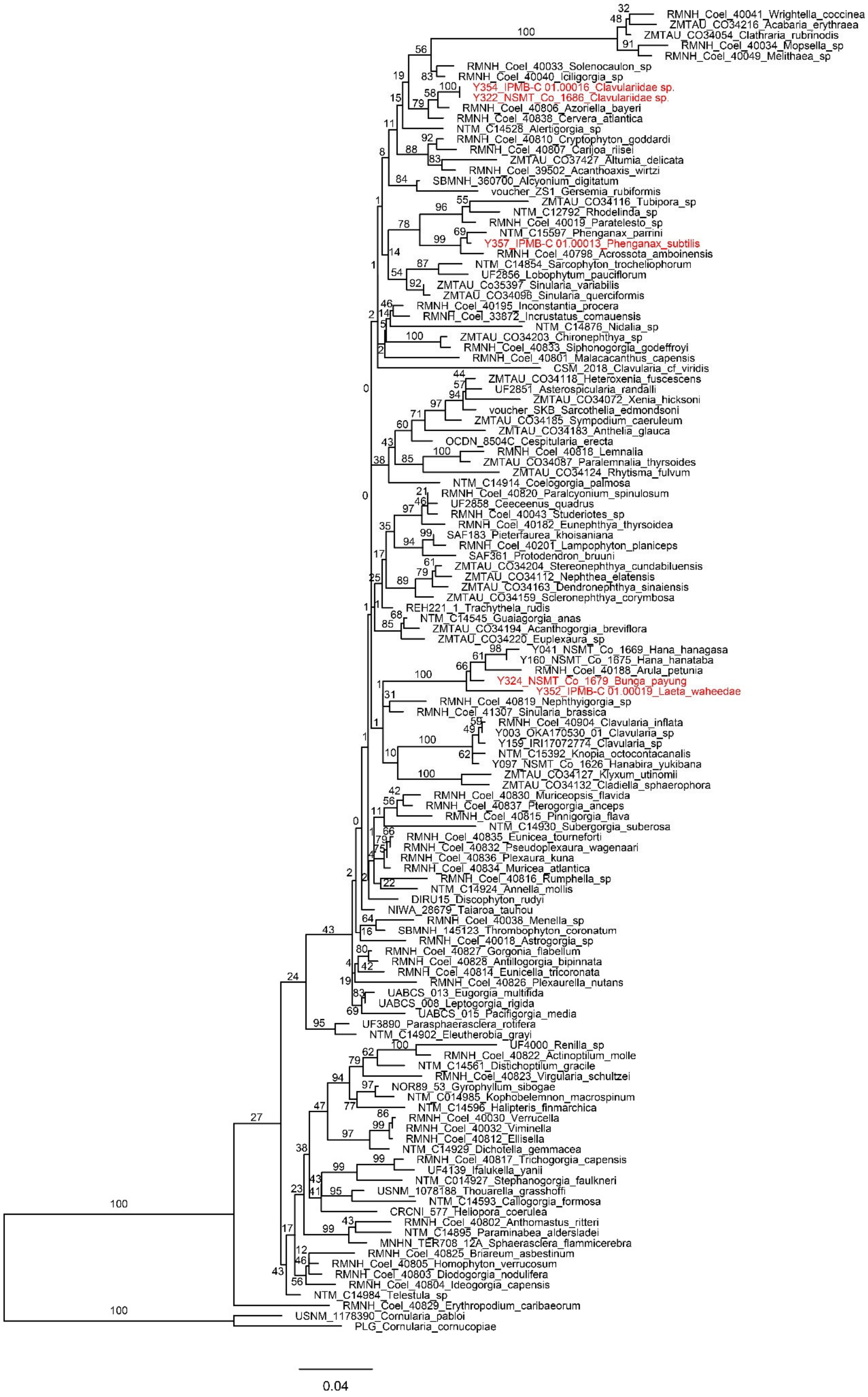

Figure S2. Maximum Likelihood phylogeny reconstruction of COI gene region of stoloniferous octocorals (Clavulariidae sp., *Bunga payung* gen. nov. et sp. nov., *Laeta waheedae* gen. nov. et sp. nov. and *Phenganax subtilis* sp. nov.) from off the coast of NW Sabah (Malaysia), and octocoral references from 121 genera, including outgroup *Cornularia* spp.

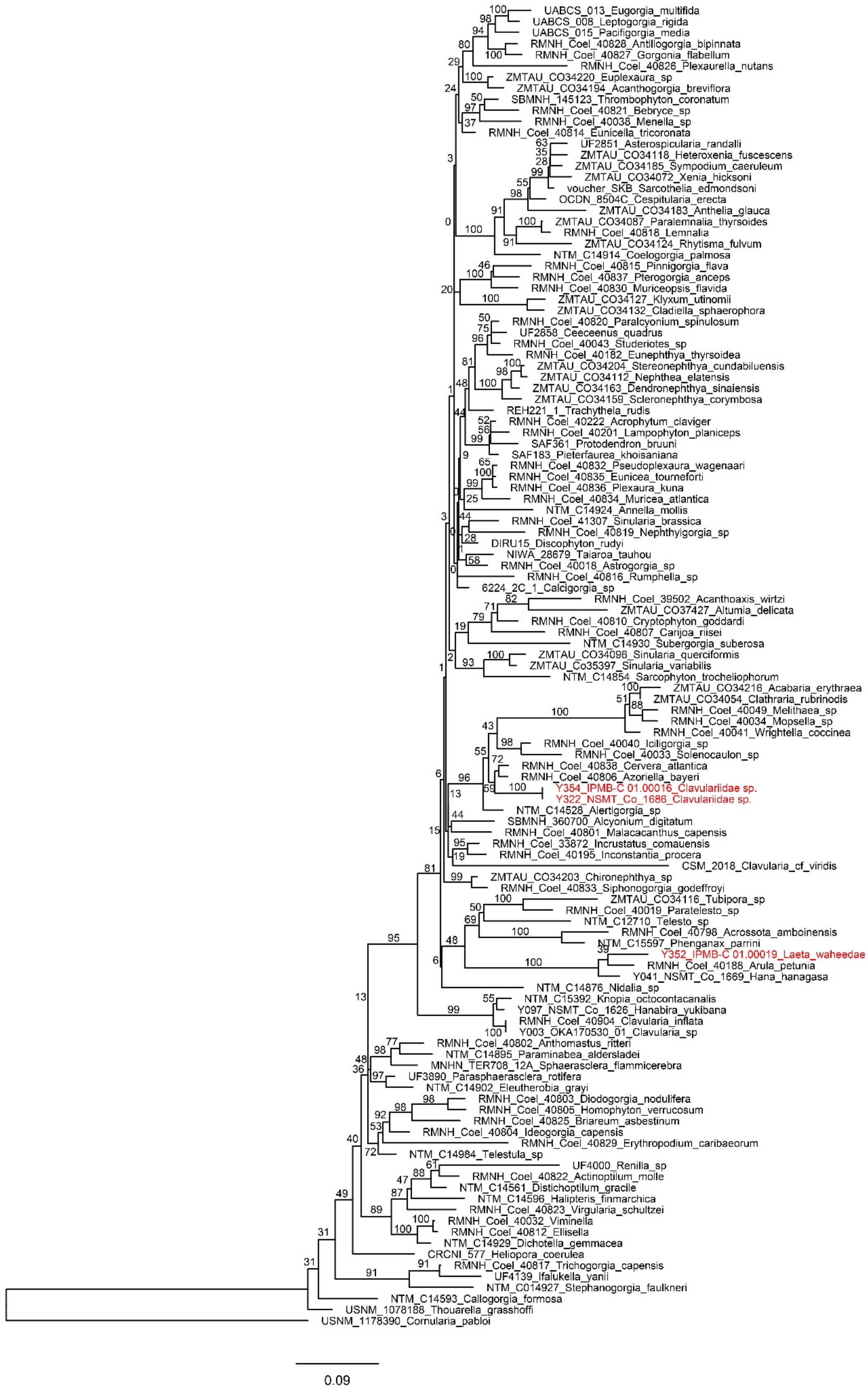

Figure S3. Maximum Likelihood phylogeny reconstruction of mtMutS gene region of stoloniferous octocorals (Clavulariidae sp. and *Laeta waheedae* gen. nov. et sp. nov.) from off the coast of NW Sabah (Malaysia), and octocoral references from 121 genera, including outgroup *Cornularia* spp.

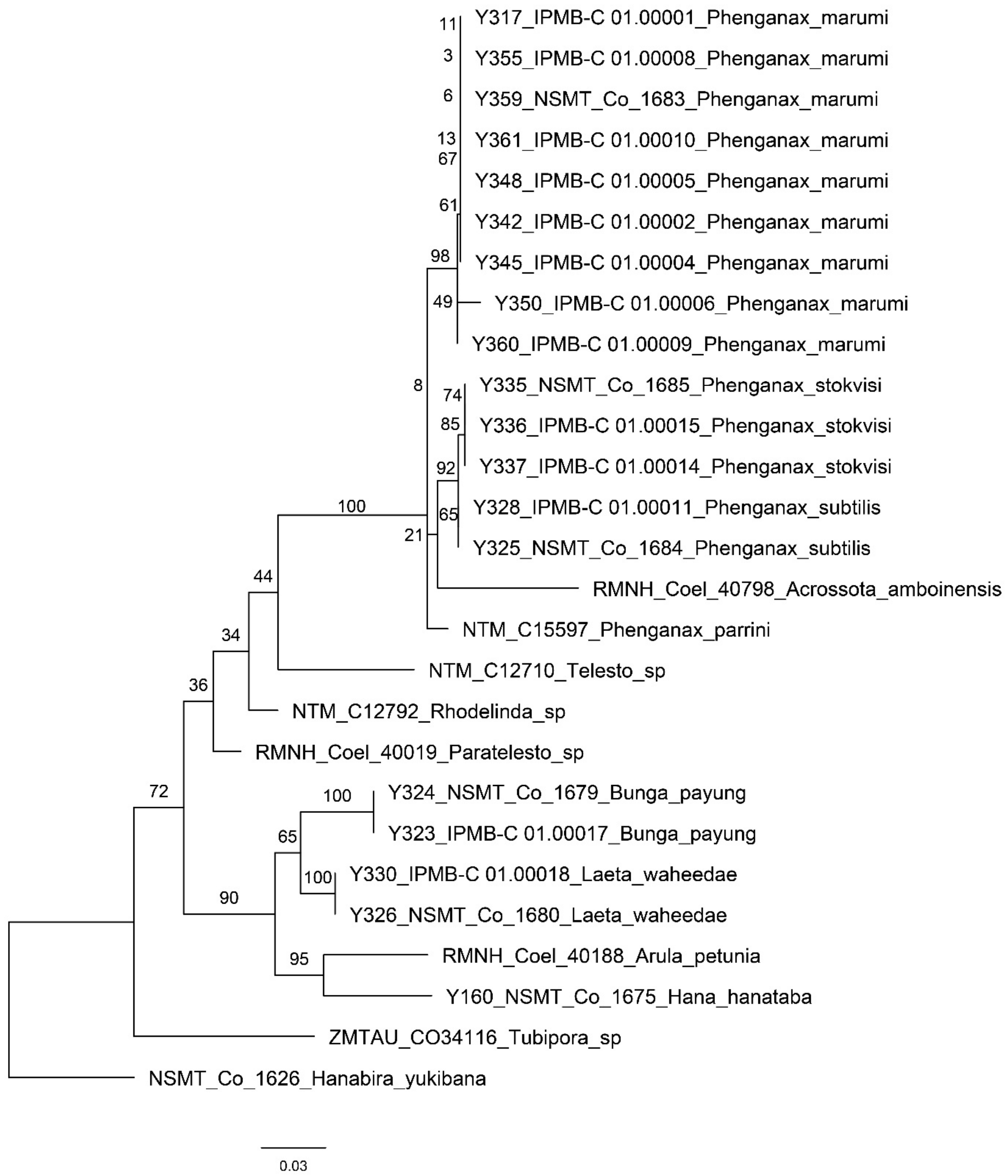

Figure S4. Maximum Likelihood phylogeny reconstruction of 28S rDNA gene region of arulid octocorals *Bunga payung* gen. nov. et sp. nov. and *Laeta waheedae* gen. nov. et sp. nov., and clavulariid octocorals, *P. marumi* sp. nov., *P. subtilis* sp. nov. and *P. stokvisi* sp. nov. from off the coast of NW Sabah (Malaysia), and nine octocoral references (*Telesto* sp., *Acrossota amboinensis*, *Rhodelinda* sp., *Tubipora* sp., *Arula petunia*, *Hana hanataba*, *Phenganax parrini*, and outgroup *Hanabira yukibana*).

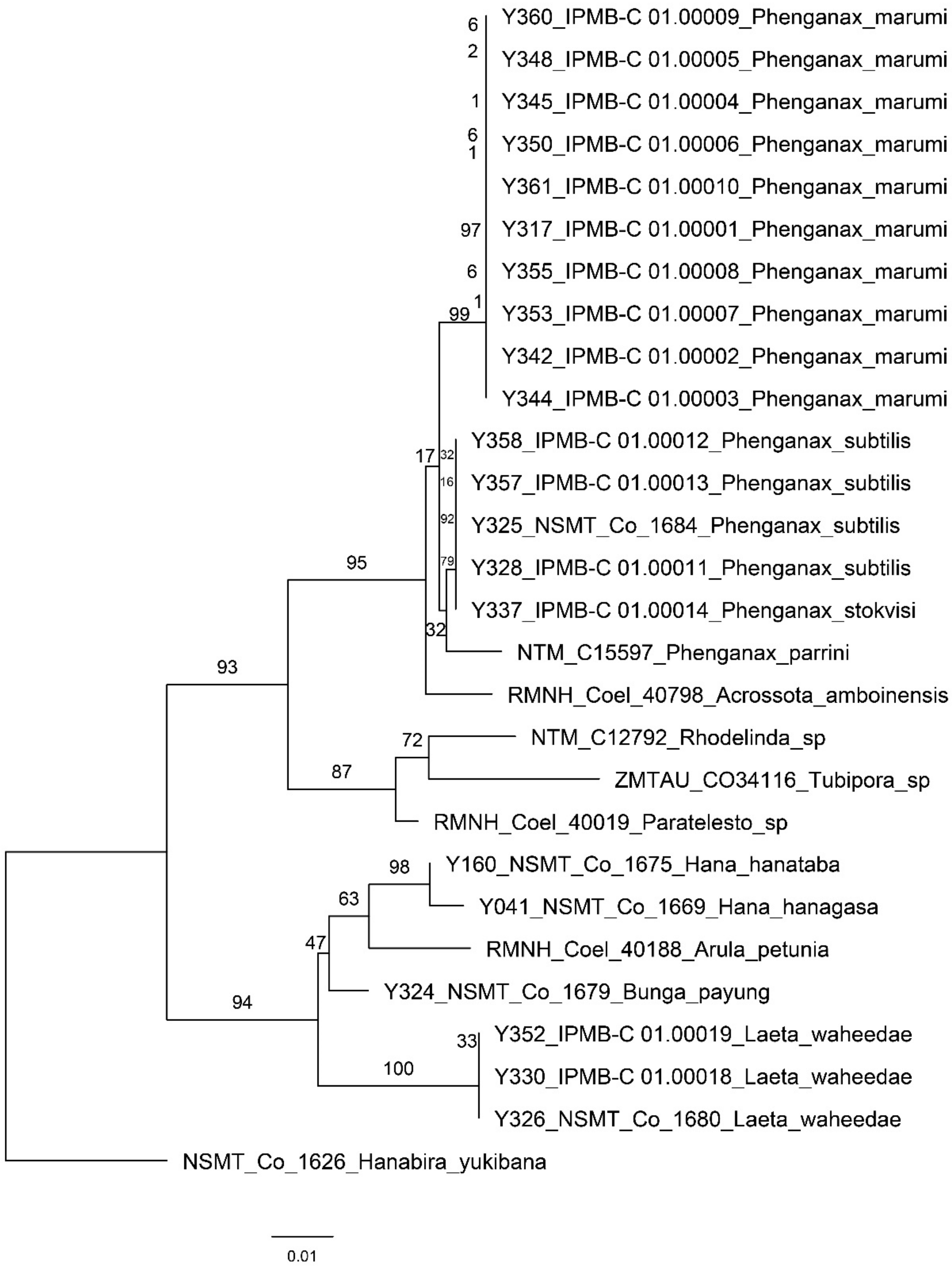

Figure S5. Maximum Likelihood phylogeny reconstruction of COI gene region of arulid octocorals *Bunga payung* gen. nov. et sp. nov. and *Laeta waheedae* gen. nov. et sp. nov., and clavulariid octocorals, *P. marumi* sp. nov., *P. subtilis* sp. nov. and *P. stokvisi* sp. nov. from off the coast of NW Sabah (Malaysia) and nine octocoral references (*Paratelesto* sp., *Acrossota amboinensis*, *Rhodelinda* sp., *Tubipora* sp., *Arula petunia*, *H. hanataba*, *H. hanagasa*, *Phenganax parrini* and outgroup *Hanabira yukibana*).

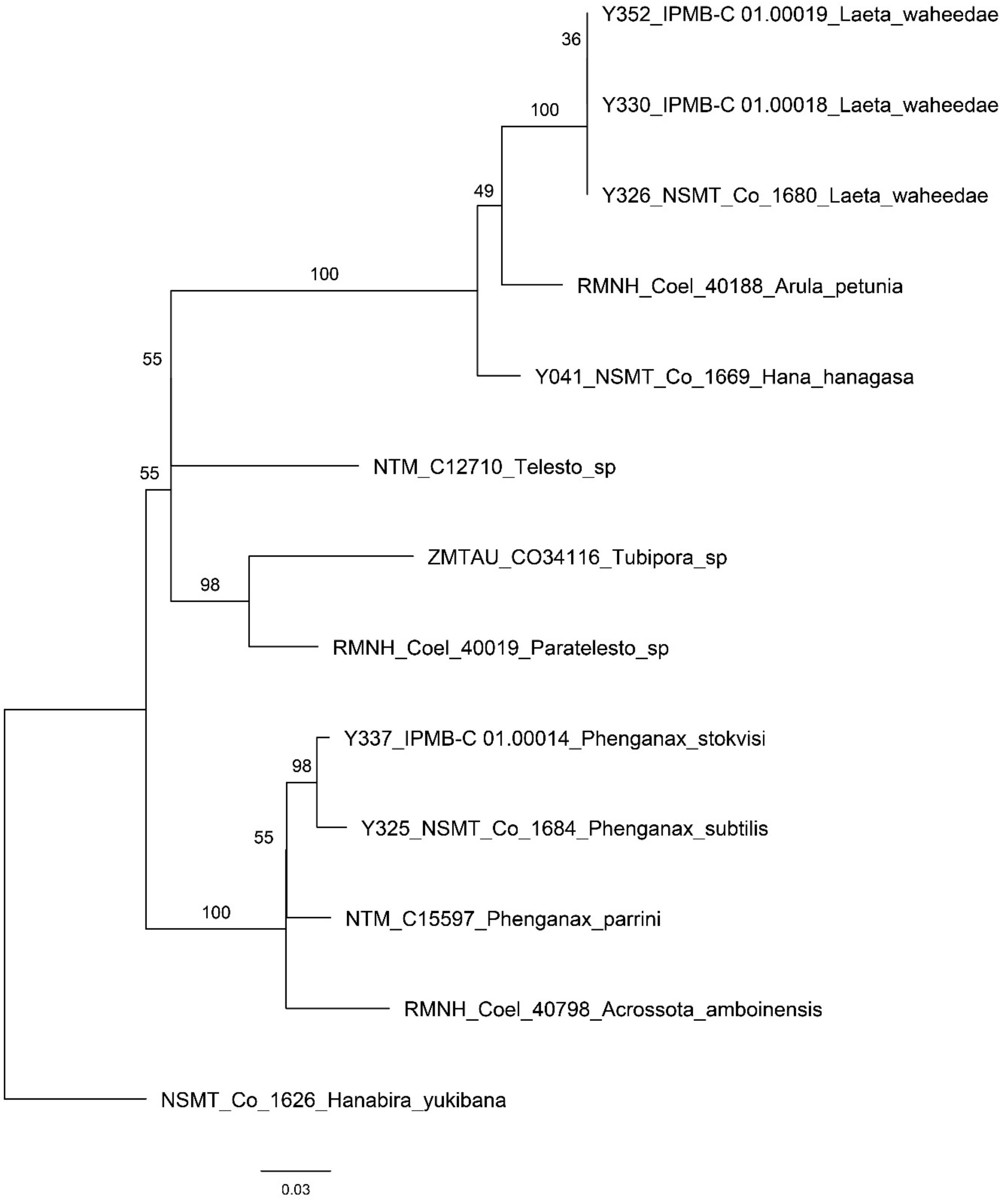

Figure S6. Maximum Likelihood phylogeny reconstruction of mtMutS gene region of arulid octocoral *Laeta waheedae* gen. nov. et sp. nov., and clavulariid octocorals, *P. subtilis* sp. nov. and *P. stokvisi* sp. nov. from off the coast of NW Sabah (Malaysia), and eight octocoral references (*Paratelesto* sp., *Telesto* sp., *Acrossota amboinensis*, *Tubipora* sp., *Arula petunia*, *H. hanagasa*, *Phenganax parrini* and outgroup *Hanabira yukibana*).

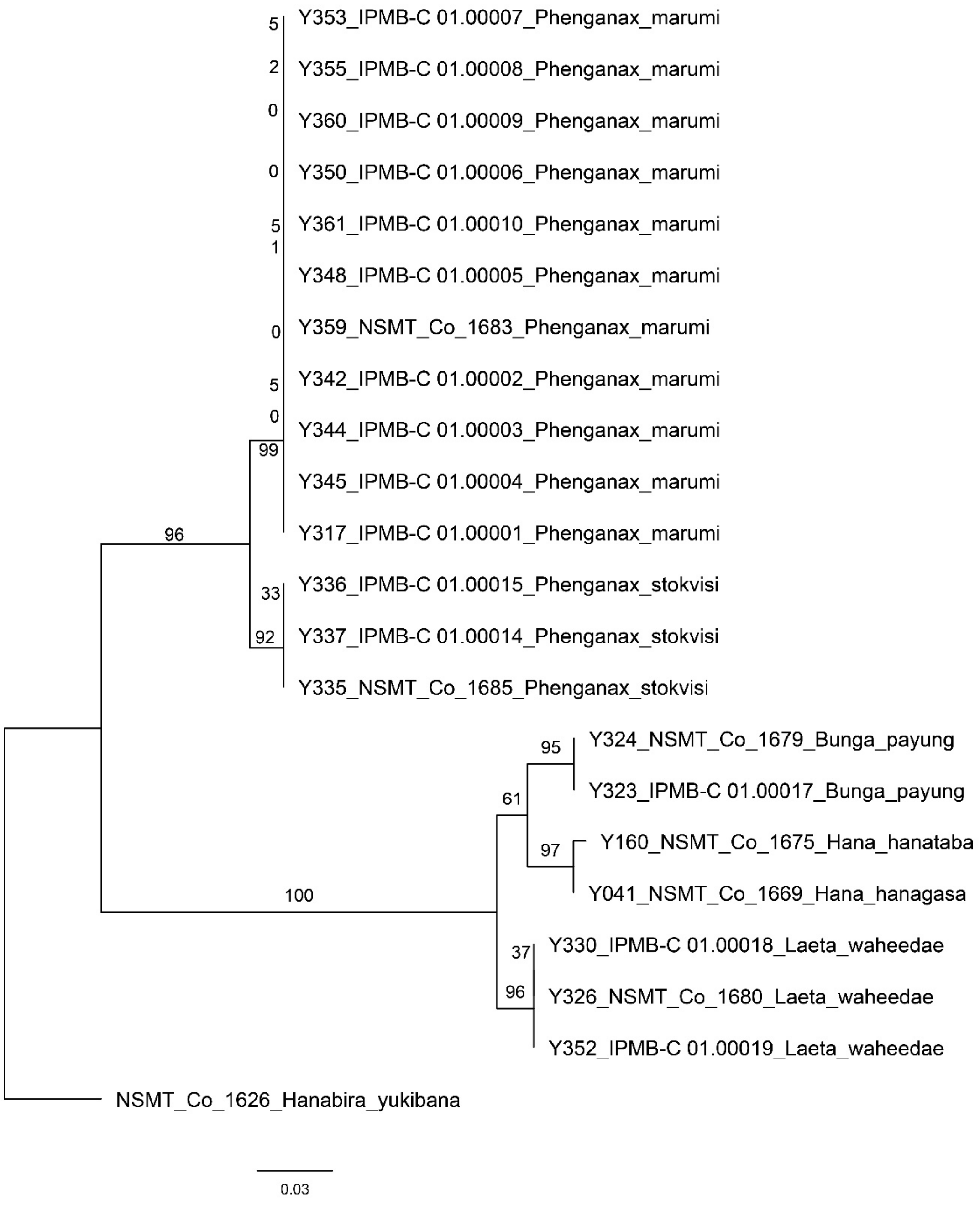

Figure S7. Maximum Likelihood phylogeny reconstruction of ND6 gene region of arulid octocorals *Bunga payung* gen. nov. et sp. nov. and *Laeta waheedae* gen. nov. et sp. nov., and clavulariid octocorals, *P. marumi* sp. nov. and *P. stokvisi* sp. nov. from off the coast of NW Sabah (Malaysia), and three octocoral references (*H. hanataba*, *H. hanagasa* and outgroup *Hanabira yukibana*).

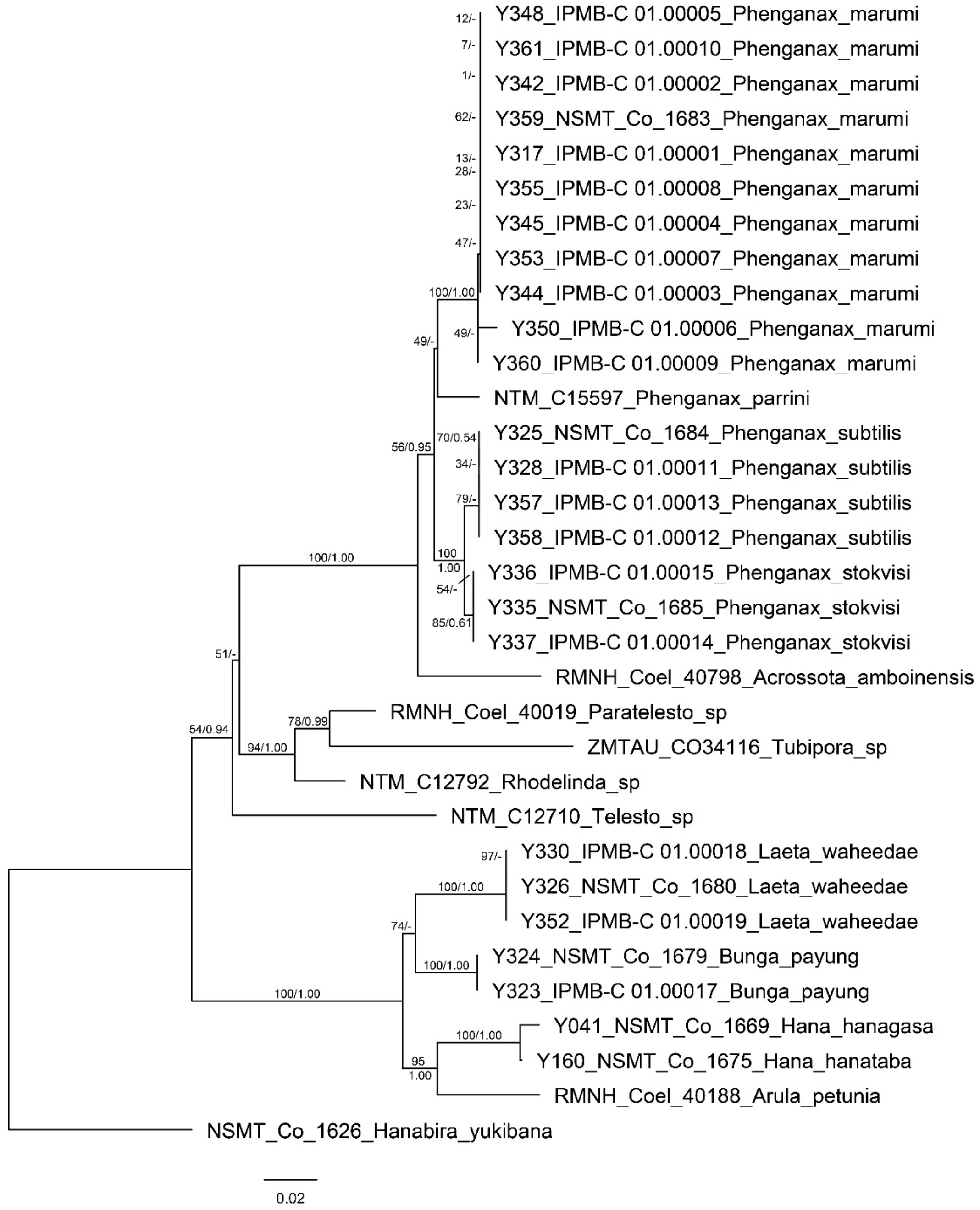

Figure S8. Combined 28S rDNA+COI+mtMutS phylogeny reconstruction for four *Phenganax* species and four arulid genera (*Hana*, *Arula*, *Bunga* gen. nov. and *Laeta* gen. nov.) collected from off the coast of NW Sabah (Malaysia); *Hanabira yukibana* was used as outgroup (total n = 33). The best Maximum Likelihood tree is shown, with values at branches representing bootstrap probabilities (left/top) and Bayesian posterior probabilities (right/bottom).

Table S1. Number of base differences per site from averaging over all sequence pairs between stoloniferous octocoral genera (*Bunga* gen. nov., *Arula*, *Hana*, *Laeta* gen. nov., *Rhodelinda*, *Paratelesto*, *Tubipora*, *Phenganax*, *Acrossota*) is shown (p expressed as percentage) for COI and mtMutS gene regions. Standard error estimates (S.E.) are shown above the diagonal. Analysis involved 37 and 18 nucleotide sequences for COI and mtMutS, respectively. All positions containing gaps and missing data were eliminated. There were totals of 717 and 709 positions in the final dataset for COI and mtMutS, respectively. Evolutionary analyses were conducted in MEGA X (Kumar et al. 2018).

**COI**	**Between groups**	**1.**	**2.**	**3.**	**4.**	**5.**	**6.**	**7.**	**8.**	**9.**
	1. *Bunga*		0.63	0.60	0.64	1.03	0.82	0.93	0.86	0.87
	2. *Arula*	2.59		0.63	0.81	1.22	0.99	1.10	0.92	0.98
	3. *Hana*	2.60	2.85		0.69	1.11	0.95	1.02	0.94	0.93
	4. *Laeta*	3.21	4.58	3.67		1.10	0.91	1.01	0.85	0.88
	5. *Rhodelinda*	6.08	7.88	7.12	7.37		0.59	0.75	0.96	0.96
	6. *Paratelesto*	5.65	7.25	7.11	6.78	2.21		0.62	0.73	0.77
	7. *Tubipora*	7.34	8.96	8.52	9.04	3.68	3.11		0.83	0.83
	8. *Phenganax*	6.44	7.26	7.52	6.35	5.79	4.27	5.45		0.44
	9. *Acrossota*	6.78	7.79	7.58	6.50	5.52	4.52	5.51	1.71	

**mtMutS**	**Between groups**	**1.**	**2.**	**3.**	**4.**	**5.**	**6.**	**7.**	**8.**
	1. *Laeta*		0.87	0.85	1.33	1.44	1.32	1.31	1.78
	2. *Hana*	6.12		0.84	1.26	1.39	1.30	1.26	1.63
	3. *Arula*	5.65	5.37		1.31	1.40	1.28	1.26	1.68
	4. *Paratelesto*	16.20	14.78	14.86		1.01	1.11	1.12	1.40
	5. *Tubipora*	19.02	18.50	17.53	8.21		1.20	1.22	1.55
	6. *Telesto*	16.34	15.33	14.86	10.70	12.46		1.17	1.52
	7. *Phenganax*	17.08	16.41	16.33	11.30	12.44	12.78		0.94
	8. *Acrossota*	20.42	17.95	19.17	12.92	13.75	14.38	5.69	

Table S2. Number of base differences per site from averaging over all sequence pairs between stoloniferous octocoral taxa (*Azoriella*, *Cervera*, *Alertigorgia*, *Solenocaulon*, *Iciligorgia*, and Clavulariidae sp.) is shown (p expressed as percentage) for gene regions COI and mtMutS. Standard error estimates (S.E.) are shown above the diagonal. Analysis involved 7 nucleotide sequences for both COI and mtMutS. All positions containing gaps and missing data were eliminated. There were totals of 717 and 881 positions in the final dataset for COI and mtMutS, respectively. Evolutionary analyses were conducted in MEGA X (Kumar et al. 2018).

**COI**	**Between groups**	**1.**	**2.**	**3.**	**4.**	**5.**	**6.**
	1. Clavulariidae sp.		0.42	0.48	0.64	0.72	0.69
	2. *Azoriella*	1.41		0.45	0.66	0.66	0.64
	3. *Cervera*	1.84	1.55		0.65	0.65	0.58
	4. *Alertigorgia*	3.39	3.67	3.25		0.72	0.65
	5. *Solenocaulon*	4.10	3.25	3.39	3.81		0.40
	6. *Iciligorgia*	3.95	3.39	2.97	3.11	1.27	

**mtMutS**	**Between groups**	**1.**	**2.**	**3.**	**4.**	**5.**	**6.**
	1. Clavulariidae sp.		0.81	7.72	1.03	1.02	0.92
	2. *Azoriella*	5.09		5.40	0.94	0.94	0.78
	3. *Cervera*	5.39	1.85		0.86	0.94	0.76
	4. *Alertigorgia*	5.94	4.30	3.71		1.11	0.94
	5. *Solenocaulon*	8.91	7.35	6.58	7.58		0.75
	6. *Iciligorgia*	7.35	5.23	4.55	4.92	4.38	

Table S3. Estimates of average evolutionary divergence over sequence pairs within stoloniferous octocoral genera for COI (*Bunga* gen. nov., *Arula*, *Hana*, *Laeta* gen. nov., *Rhodelinda*, *Paratelesto*, *Tubipora*, *Phenganax*, *Acrossota*) and mtMutS (*Arula*, *Hana*, *Laeta*, *Rhodelinda*, *Paratelesto*, *Tubipora*, *Phenganax*, *Acrossota*) gene regions. The numbers of base differences per site from averaging over all sequence pairs within each group (d) are shown (uncorrected *p*, expressed as percentage). Standard error estimates (S.E.) are shown in the second column and were obtained by a bootstrap procedure (1000 replicates). Analyses involved 37 and 18 nucleotide sequences for COI and mtMutS, respectively. All positions containing gaps and missing data were eliminated. There were totals of 717 and 709 positions in the final dataset for COI and mtMutS, respectively. Evolutionary analyses were conducted in MEGA X (Kumar et al. 2018).

	**COI**			**mtMutS**	
**Within groups**	**d**	**S.E.**	**Within groups**	**d**	**S.E.**
1. *Bunga*	n/c	n/c	1. *Laeta*	0.00	0.00
2. *Arula*	0.00	0.00	2. *Hana*	0.37	0.17
3. *Hana*	0.27	0.13	3. *Arula*	0.00	0.00
4. *Laeta*	0.00	0.00	4. *Paratelesto*	n/c	n/c
5. *Rhodelinda*	n/c	n/c	5. *Tubipora*	n/c	n/c
6. *Paratelesto*	n/c	n/c	6. *Telesto*	n/c	n/c
7. *Tubipora*	n/c	n/c	7. *Phenganax*	3.10	0.52
8. *Phenganax*	0.57	0.18	8. *Acrossota*	n/c	n/c
9. *Acrossota*	n/c	n/c			

Table S4. Estimates of average evolutionary divergence over sequence pairs within stoloniferous octocoral taxa for COI (*Azoriella*, *Cervera*, *Alertigorgia*, *Solenocaulon*, *Iciligorgia*, and Clavulariidae sp.) and mtMutS (Clavulariidae sp.) gene regions. The numbers of base differences per site from averaging over all sequence pairs within each group (d) are shown (uncorrected *p*, expressed as percentage). Standard error estimates (S.E.) are shown in the second column and were obtained by a bootstrap procedure (1000 replicates). Analyses involved 7 nucleotide sequences for both COI and mtMutS. All positions containing gaps and missing data were eliminated. There were totals of 717 and 881 positions in the final dataset for COI and mtMutS, respectively. Evolutionary analyses were conducted in MEGA X (Kumar et al. 2018).

	**COI**			**mtMutS**	
**Within groups**	**d**	**S.E.**	**Within groups**	**d**	**S.E.**
1. Clavulariidae sp.	0.00	0.00	1. Clavulariidae sp.	0.00	0.00
2. *Azoriella*	n/c	n/c	2. *Azoriella*	n/c	n/c
3. *Cervera*	n/c	n/c	3. *Cervera*	n/c	n/c
4. *Alertigorgia*	n/c	n/c	4. *Alertigorgia*	n/c	n/c
5. *Solenocaulon*	n/c	n/c	5. *Solenocaulon*	n/c	n/c
6. *Iciligorgia*	n/c	n/c	6. *Iciligorgia*	n/c	n/c

Table S5. Number of base differences per site from averaging over all sequence pairs between stoloniferous octocoral species is shown (*p* expressed as percentage) for COI (*H. hanagasa*, *H. hanataba*, *P. marumi* sp. nov., *P. subtilis* sp. nov., *P. parrini*, *P. stokvisi* sp. nov.) and mtMutS (*H. hanagasa*, *H. hanataba*, *P. subtilis* sp. nov., *P. parrini*, *P. stokvisi* sp. nov.) gene regions. Standard error estimates (S.E.) are shown above the diagonal. Analysis involved 28 and 9 nucleotide sequences for COI and mtMutS, respectively. All positions containing gaps and missing data were eliminated. There were totals of 717 and 709 positions in the final dataset for COI and mtMutS, respectively. Evolutionary analyses were conducted in MEGA X (Kumar et al. 2018).

**COI**	**Between groups**	**1.**	**2.**	**3.**	**4.**	**5.**	**6.**
	1. *H. hanagasa*		0.28	0.95	0.95	0.96	1.37
	2. *H. hanataba*	0.56		0.93	0.94	0.95	1.32
	3. *P. marumi*	7.64	7.34		0.39	0.47	0.85
	4. *P. subtilis*	7.49	7.20	0.98		0.37	0.79
	5. *P. parrini*	7.91	7.63	1.69	0.99		0.90
	6. *P stokvisi*	10.00	9.32	3.64	3.18	4.09	

**mtMutS**	**Between groups**	**1.**	**2.**	**3.**	**4.**	**5.**
	1. *H. hanagasa*		0.30	1.29	1.26	1.33
	2. *H. hanataba*	0.67		1.28	1.26	1.33
	3. *P. subtilis*	16.57	16.64		0.46	0.3
	4. *P. stokvisi*	15.72	15.79	1.60		0.70
	5. *P. parrini*	16.87	16.94	4.07	3.64	

Table S6. Estimates of average evolutionary divergence over sequence pairs within stoloniferous octocoral species for COI (*H. hanagasa*, *H. hanataba*, *P. marumi*, *P. subtilis*) and mtMutS (*H. hanagasa*, *H. hanataba*) gene regions. The numbers of base differences per site from averaging over all sequence pairs within each group (d) are shown (uncorrected *p*, expressed as percentage). Standard error estimates (S.E.) are shown in the second column and were obtained by a bootstrap procedure (1000 replicates). Analyses involved 28 and 9 nucleotide sequences for COI and mtMutS, respectively. All positions containing gaps and missing data were eliminated. There were totals of 717 and 709 positions in the final dataset for COI and mtMutS, respectively. Evolutionary analyses were conducted in MEGA X (Kumar et al. 2018).

	**COI**			**mtMutS**	
**Within groups**	**d**	**S.E.**	**Within groups**	**d**	**S.E.**
1. *H. hanagasa*	0.00	0.00	1. *H. hanagasa*	0.00	0.00
2. *H. hanataba*	0.00	0.00	2. *H. hanataba*	0.15	0.14
3. *P. marumi*	0.00	0.00	4. *P. subtilis*	n/c	n/c
4. *P. subtilis*	0.00	0.00	5. *P. parrini*	n/c	n/c
5. *P. parrini*	n/c	n/c	6. *P. stokvisi*	n/c	n/c
6. *P. stokvisi*	n/c	n/c			
